# Examination of overall treatment effect and the proportion attributable to contextual effect in osteoarthritis: meta-analysis of randomised controlled trials

**DOI:** 10.1136/annrheumdis-2015-208387

**Published:** 2016-02-16

**Authors:** Kun Zou, Jean Wong, Natasya Abdullah, Xi Chen, Toby Smith, Michael Doherty, Weiya Zhang

**Affiliations:** 1 Division of Rheumatology, Orthopaedics and Dermatology, University of Nottingham, Nottingham, UK; 2 Sichuan Academy of Medical Sciences & Sichuan Provincial People's Hospital, Affiliated Hospital of University of Electronic Science and Technology, Chengdu, China; 3 Pinfold Medical Practice, Loughborough, UK; 4 School of Health Sciences, University of East Anglia, Norwich, UK

**Keywords:** Osteoarthritis, Treatment, Epidemiology

## Abstract

**Objective:**

To examine the overall treatment effect and the proportion attributable to contextual effect (PCE) in randomised controlled trials (RCTs) of diverse treatments for osteoarthritis (OA).

**Methods:**

We searched Medline, Embase, Central, Science Citation Index, AMED and CINAHL through October 2014, supplemented with manual search of reference lists, published meta-analyses and systematic reviews. Included were RCTs in OA comparing placebo with representative complementary, pharmacological, non-pharmacological and surgical treatments. The primary outcome was pain. Secondary outcomes were function and stiffness. The effect size (ES) of overall treatment effect and the PCE were pooled using random-effects model. Subgroup analyses and meta-regression were conducted to examine determinants of the PCE.

**Results:**

In total, 215 trials (41 392 participants) were included. The overall treatment effect for pain ranged from the smallest with lavage (ES=0.46, 95% CI 0.24 to 0.68) to the largest with topical non-steroidal anti-inflammatory drugs (ES=1.37, 95% CI 1.19 to 1.55). On average, 75% (PCE=0.75, 95% CI 0.72 to 0.79) of pain reduction was attributable to contextual effect. It varied by treatment from 47% (PCE=0.47, 95% CI 0.32 to 0.70) for intra-articular corticosteroid to 91% (PCE=0.91, 95% CI 0.60 to 1.37) for joint lavage. Similar results were observed for function and stiffness. Treatment delivered by needle/injection and other means than oral medication, longer duration of treatment, large sample size (≥100 per arm) and public funding source were associated with increased PCE for pain reduction.

**Conclusions:**

The majority (75%) of the overall treatment effect in OA RCTs is attributable to contextual effects rather than the specific effect of treatments. Reporting overall treatment effect and PCE, in addition to traditional ES, permits a more balanced, clinically meaningful interpretation of RCT results. This would help dispel the frequent discordance between conclusions from RCT evidence and clinical experience—the ‘efficacy paradox’.

## Introduction

The benefit of a treatment may result from the specific effect of the treatment itself and the non-specific effect from the context in which the treatment is delivered.^1^ This non-specific effect is commonly termed as placebo effect in clinical trials,^2^ and placebo response or contextual effect in clinical practice.^3^ However, the clinical impact of the latter has largely been overlooked, especially since the placebo effect in a randomised controlled trial (RCT) is usually subtracted from the treatment effect. In this situation, a treatment is only considered effective when it is clearly superior to placebo, and it is this difference from placebo that is reported in terms of the strength of the treatment. However, in clinical practice, a treatment is unavoidably delivered with contextual factors and it is the overall effect of the treatment (specific plus contextual effects) that is important to the patient. A large number of studies have demonstrated that contextual factors such as patient beliefs and expectancy, the patient–practitioner interaction and the environment have real therapeutic effects.^3–5^ These benefits are often clinically significant, especially in chronically painful or distressing conditions.^2^ ^6^

Osteoarthritis (OA) is the most common form of arthritis. In the USA, it affects 27 million people or 12.1% of the adult population.^7^ ^8^ Current treatments mainly aim to relieve pain and stiffness and to improve function and quality of life.^9^ However, the benefits from current available therapies are relatively small and these may be outweighed by side effects and other factors such as cost of delivery.^10^ Of 53 treatments, only two (opioid and intra-articular corticosteroid injection) have been reported to consistently reach the minimum clinically important difference (MCID) with an effect size (ES) of 0.5 over placebo.^9^ ^10^ This is equivalent to 15% of pain reduction on a visual analogue scale (VAS).^11^ Although the magnitude of an acceptable MCID continues to be debated, it is apparent that the additional benefit of treatment above placebo is not the only benefit that a patient receives from a treatment, both in RCTs and in clinical practice. The sole focus on the separation of treatment from placebo causes confusion to practitioners when a treatment reported to have a small ES in an RCT clearly produces clinically important improvements in clinical practice. Such common discordance between reported small treatment effects in RCTs and guidelines and the observed marked treatment effects in clinical practice presents an ‘efficacy paradox’ to many patients and clinicians.^12^ This suggests the need for a change in emphasis in the reporting and interpretation of results of placebo-controlled RCTs. Based on this, this study examined the overall treatment effect in RCTs and the proportion of that effect that may be explained by placebo, rather than conventional separation of treatment from placebo, in an attempt to overcome this ‘efficacy paradox’. For this first exploration, we sampled specific interventions aimed at managing OA, with different models of action and delivery, including pharmaceutical, non-pharmaceutical, surgical and complementary treatments, rather than examine all treatments for OA.

## Methods

A systematic review (SR) and meta-analysis of randomised placebo-controlled trials was performed.

### Search strategy and selection criteria

A systematic search was undertaken using the Cochrane Library, Medline (OVID), Embase (OVID), Web of Science, AMED and CINAHL from inception to October 2014. Free texts and index terms related to ‘osteoarthritis’, ‘randomised controlled trial’ and a specific treatment (eg, paracetamol or acetaminophen) were used (see online supplementary search strategy). Reference lists of included studies and published SRs and meta-analyses were hand searched for additional eligible studies. No language limitation was applied.

Studies meeting the following criteria were included: (1) randomised placebo-controlled trial; (2) participants with OA of any joint; (3) comparisons of placebo with active treatment including chondroitin, glucosamine, paracetamol, oral non-steroidal anti-inflammatory drugs (NSAIDs), topical NSAIDs, pulsed electromagnetic field therapy (PEMF), acupuncture, intra-articular hyaluronic acid (IAHA), intra-articular corticosteroid (IACS) and joint lavage; (4) reporting at least one of the following outcomes: pain, function or stiffness; and (5) reporting change from baseline and its SD or data that could derive them.

### Data extraction and quality assessment

A standard data form was used to extract data of included studies. Items recorded were study design and setting, characteristics of participants (percentage of women, mean age), interventions (sessions, duration) and outcomes (at different time points). Repeated measurements of change from baseline and its SD were collected. If not presented, they were calculated from outcomes at baseline and end points using a formula recommend by the Cochrane Collaboration, that SD of the change was adjusted for the correlation between baseline and endpoint values.^13^ ^14^ The correlation coefficient was obtained from trials that reported SD at both baseline, end point and of the change from baseline. When more than one scale for the same outcome was reported, for example, Western Ontario and McMaster Universities Arthritis Index pain and VAS pain, only one scale per outcome was selected using a published outcome measure hierarchy.^15^

Study quality was assessed using the modified Jadad tool in which allocation concealment was also assessed.^13^ ^16^ Data were fully extracted and assessed by a single investigator (KZ) and validated by three other investigators (NA, XC and TS). Discrepancies were discussed and ratified by a senior investigator (WZ).

### Statistical analysis

The overall treatment effect was defined as the ES of active treatment group, whereas the contextual effect was defined as the ES of the placebo group. The ES in terms of mean change from baseline in the unit of its SD was calculated for each group.^14^ The proportion attributable to contextual effect (PCE) and its 95% CI were calculated using the ES ratio between the contextual effect and the overall treatment effect.^17^ Theoretically, the PCE should range from 0 (which indicated no contribution from contextual effects) to 1 (which indicated 100% contribution from contextual effects). When the ES of contextual effects was greater than the ES of overall treatment effects, the maximum of 1 (100%) was given. Trials in which patients in either treatment or placebo group worsened from baseline were excluded from the meta-analysis since (1) it may be side/nocebo effect, which is not the focus of interest for the PCE; and (2) the measure of PCE does not allow negative values, especially when the ratio was log transformed.

The primary outcome measure was pain. Secondary outcome measures were function and stiffness. The time point when the ES of active treatment group reached its peak in each study was chosen for meta-analyses. The heterogeneity of studies was assessed using Q test and I^2^ index tests.^13^ ^18^ Publication bias was accessed using funnel plot and Egger's test.^19^ Random-effects model was applied in all meta-analyses to account for potential heterogeneity.

Subgroup analyses were performed to assess the effect of type of treatment; sample size (≥100 per arm);^20^ duration of intervention (at 4, 8, 13 and >13 weeks); route of treatment (oral, PEMF, topical, needle/injection or surgery); chance of receiving active treatment (number of active treatment arms/number of treatment arms); allocation concealment (yes vs no); blinding of participants (yes vs no) and setting of trials (primary care vs secondary care); funding source (public vs industry); and targeted joint and country (developing vs developed). Random-effect meta-regression was also conducted to assess the potential determinants of the PCE for pain. All statistical tests were performed using STATA V.11 (Stata Corp LP, Texas, USA).

## Results

### Study selection

**Figure 1:**
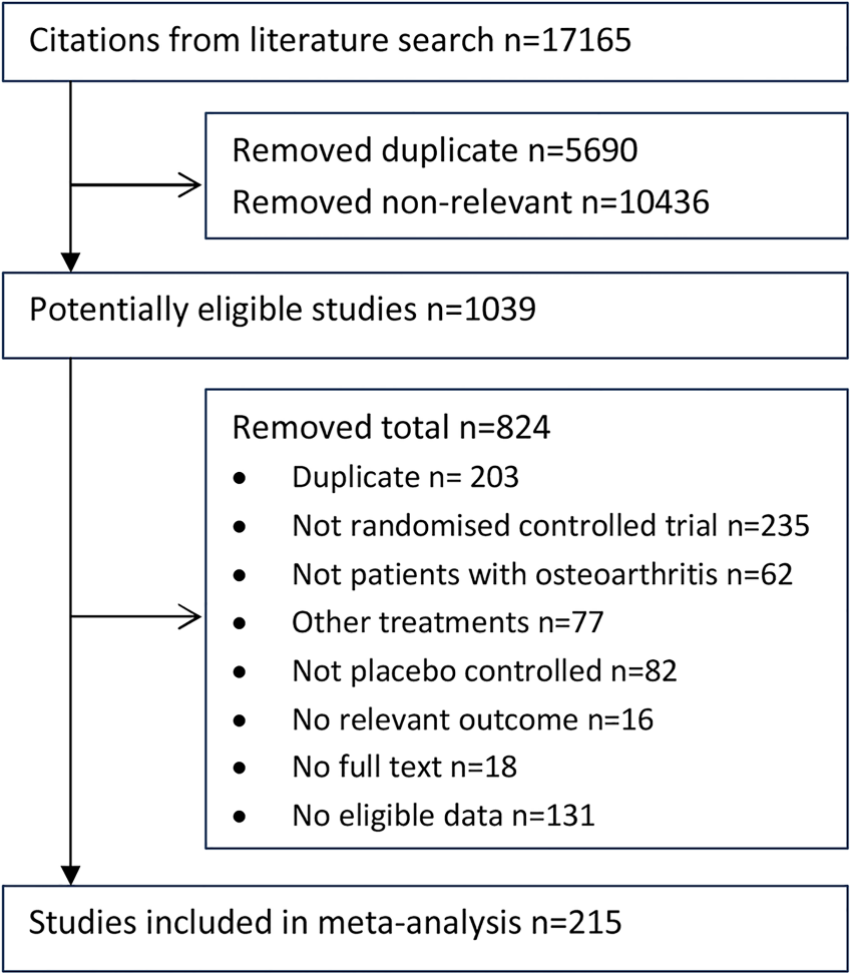
Flow chart of study selection.

The literature search identified 17 165 citations. After initial screening of titles and abstracts, 1039 potentially eligible citations were identified. Of those, 824 citations were excluded after reading full papers. Finally, 215 studies were included in the meta-analysis (figure 1).

### Characteristics of included studies

**Figure 2:**
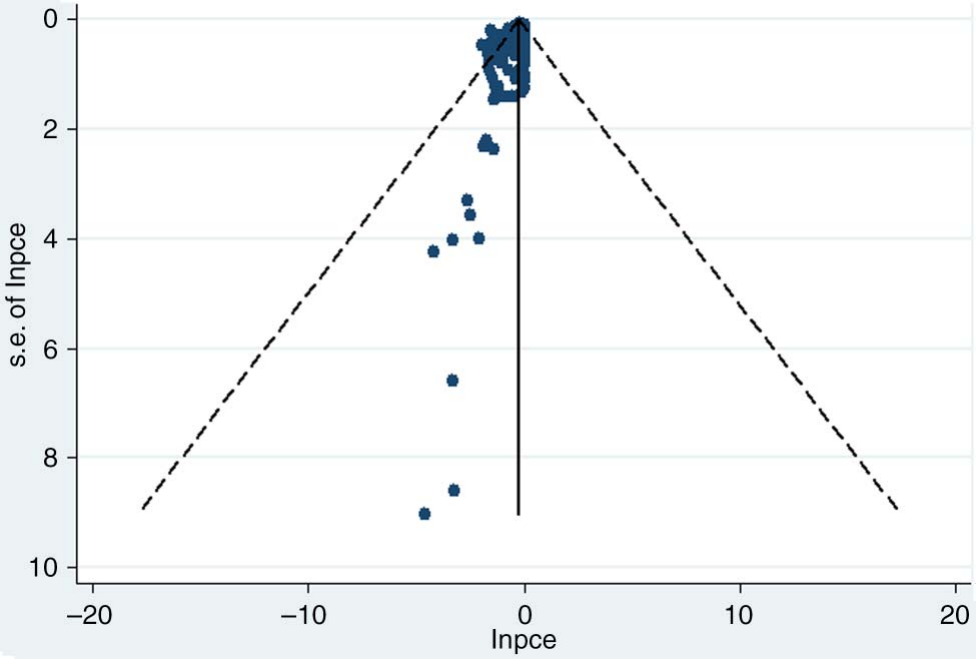
Funnel plot of LnPCE for pain in osteoarthritis. PCE, proportion attributable to contextual effect, Egger test p<0.001.

From the 215 studies, 41 392 participants were included in the analysis. The median age of patients was 62.2 (IQR 60.0 to 64.2) years; median percentage of women was 65.8% (IQR 60.0% to 72.8%); pooled baseline pain was 54.8 (95% CI 50.8 to 58.9) on the 0–100 scale; median duration of symptoms was 6.8 (IQR 5.0 to 8.7) years; and the median duration of study was 12 (IQR 4 to 13) weeks. The main methodological limitation was lack of allocation concealment, which was the case in about 50% (109/215) of trials. Details of summarised study characteristics by treatment are shown in table 1. Publication bias was apparent (Egger test p<0.001), that is, trials with smaller PCE (larger difference between treatment and placebo groups) were more likely to be published, which generally had a smaller sample size (figure 2). This was coherent with the funnel plot of standardised mean difference (SMD) of treatment over placebo, which showed that trials with larger SMD between the two groups were more likely to be published (see online supplementary figure S1).

**Table 1:** Summary of characteristics of included studies

	Total	Chondroitin	Glucosamine	Chondroitin + glucosamine	Paracetamol	NSAIDs	Topical NSAIDs	PEMF	Acupuncture	IACS	IAHA	Joint lavage
No. of trials	215	13	21	5	7	63	20	19	17	10	36	4
No. of participants	41 392	2546	2869	1055	2345	18 617	4400	1002	2723	612	4796	427
Median age (IQR), years	62.2 (60.0 to 64.2)	63.0 (60.0 to 65.0)	61.7 (58.3 to 64.5)	58.6 (56.7 to 67.6)	62.6 (61.9 to 64.1)	61.6 (60.1 to 62.6)	63.2 (61.5 to 65.3)	61.6 (59.6 to 63.9)	64.8 (62.8 to 67.0)	64.9 (63.2 to 66.0)	62.6 (59.5 to 64.6)	57.1 (54.0 to 60.7)
Median percentage of women (IQR), %	65.8 (60.0 to 72.8)	65.9 (55.8 to 72.1)	64.4 (60.3 to 81.3)	82.5 (70.8 to 88.2)	66.9 (64.5 to 74.8)	66.8 (62.9 to 71.0)	65.0 (61.6 to 74.5)	71.6 (61.8 to 90.2)	65.0 (61.0 to 69.1)	67.5 (61.7 to 71.4)	61.0 (51.3 to 70.1)	58.7 (29.9 to 67.2)
Median years of symptom (IQR)	6.8 (5.0 to 8.7)	6.6 (6.4 to 6.8)	9.2 (7.1 to 12.0)	7.8 (5.5 to 10.0)	7.8 (5.3 to 8.7)	7.2 (5.4 to 8.8)	7.4 (3.6 to 10.8)	6.0 (3.6 to 8.2)	7.0 (6.5 to 7.5)	7.6 (6.0 to 9.3)	4.8 (2.7 to 6.2)	10.4 (–)
Pooled baseline pain (95% CI) in 0–100 scale	54.8 (50.8 to 58.9)	57.2 (48.9 to 65.5)	51.4 (39.8 to 63.0)	42.8 (25.8 to 59.9)	49.7 (33.9 to 65.5)	56.1 (46.8 to 65.3)	59.7 (53.8 to 65.5)	55.4 (47.3 to 63.5)	41.4 (34.8 to 47.9)	60.0 (46.6 to 73.3)	56.4 (52.0 to 60.8)	65.4 (43.3 to 87.4)
Median duration of trial (IQR), weeks	12 (4 to 13)	26 (13 to 52)	12 (7 to 24)	16 (12 to 24)	6 (6 to 12)	6 (4 to 12)	4 (2 to 12)	6 (3 to 10)	9 (4 to 13)	9 (3 to 12)	16 (9 to 26)	52 (38 to 78)
Quality of included studies												
Randomisation	122	9	15	3	2	24	19	12	12	4	18	4
Allocation concealment	96	8	11	3	1	18	15	12	9	2	14	3
Blinding to participants	199	13	20	5	7	61	18	17	16	9	30	3
Intent to treat analysis	141	9	13	2	7	45	19	7	9	6	22	2

IACS, intra-articular corticosteroid; IAHA, intra-articular hyaluronic acid; NSAIDs, non-steroidal anti-inflammatory drugs; PEMF, pulsed electromagnetic field therapy.

### Overall treatment effect and the PCE

**Figure 3:**
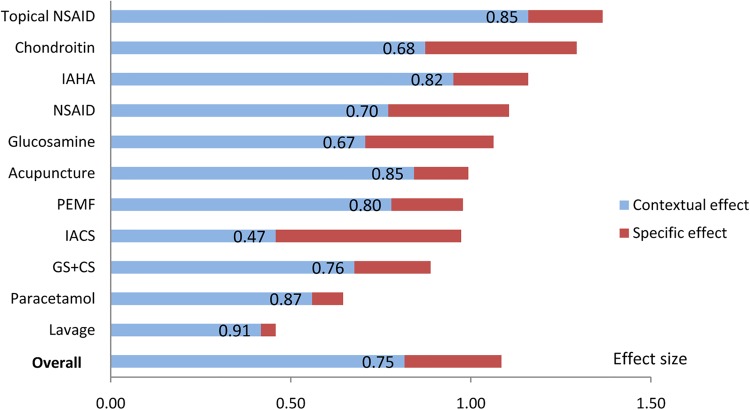
The overall treatment effect and the proportion attributable to contextual effect (PCE) for pain in osteoarthritis. The overall length of the bar represents the effect size (ES) of overall treatment effect; the blue component of the bar and the label represents the PCE of each treatment (measured by ratio of the ESs between contextual effect and overall treatment effect, ranging from no contribution from contextual effect (=0) to 100% contribution from contextual effect (=1); the red component represent the proportion attributable to specific effect of each treatment). CS, chondroitin sulfate; GS, glucosamine sulfate; IACS, intra-articular corticosteroid; IAHA, intra-articular hyaluronic acid; NSAID, non-steroidal anti-inflammatory drug; PEMF, pulsed-electromagnetic field therapy.

Of the 11 selected treatments, the overall treatment effect for pain was smallest with lavage (ES=0.46, 95% CI 0.24 to 0.68) and largest with topical NSAIDs (ES=1.37, 95% CI 1.19 to 1.55) (table 2), the PCE in these two treatments being 91% and 85%, respectively. On average, 75% of the overall treatment effects for pain in OA were explained by contextual effects (PCE=0.75, 95% CI 0.72 to 0.79) (table 2). The PCE, from the highest to the lowest, was 0.91 (95% CI 0.60 to 0.137) for lavage, 0.87(95% CI 0.73 to 1.03) for paracetamol, 0.85 (95% CI 0.77 to 0.93) for topical NSAIDs, 0.85 (95% CI 0.74 to 0.97) for acupuncture, 0.82 (95% CI 0.75 to 0.90) for IAHA, 0.80 (95% CI 0.64 to 0.99) for PEMF, 0.76 (95% CI 0.62 to 0.93) for glucosamine plus chondroitin, 0.70 (95% CI 0.65 to 0.75) for NSAIDs, 0.68 (95% CI 0.55 to 0.84) for chondroitin, 0.67 (95% CI 0.53 to 0.84) for glucosamine, and 0.47 (95% CI 0.32 to 0.70) for IACS. Figure 2 presents the hierarchy of the overall treatment effect for each active treatment and their PCE. Similar results were observed for function and stiffness (table 2). Four studies of pain and one study of stiffness in which placebo group worsened were excluded from meta-analyses (figure 3).

**Table 2:** Overall treatment effect size (ES) and proportion attributable to contextual effects (PCEs)

Outcome	Treatment	No. of trials	No. of patients	Overall treatment ES (95% CI)	I^2^% of overall treatment ES	PCE (95% CI)	I^2^% of PCE
Pain	*Oral*						
	Glucosamine	19	2512	1.06 (0.85 to 1.28)	88	0.67 (0.53 to 0.84)	63
	Chondroitin	13	2562	1.29 (1.09 to 1.50)	85	0.68 (0.55 to 0.84)	67
	Glucosamine + chondroitin	4	778	0.89 (0.27 to 1.51)	92	0.76 (0.62 to 0.93)	0
	Paracetamol	7	2377	0.65 (0.46 to 0.83)	87	0.87 (0.73 to 1.03)	12
	NSAID	62	18 145	1.11 (1.02 to 1.20)	92	0.70 (0.65 to 0.75)	51
	*Physical/topical*						
	PEMF	18	1005	0.98 (0.75 to 1.20)	78	0.80 (0.64 to 0.99)	0
	Topical NSAIDs	20	4399	1.37 (1.19 to 1.55)	90	0.85 (0.77 to 0.93)	35
	*Needle/injection*						
	Acupuncture	17	2747	0.99 (0.81 to 1.18)	85	0.85 (0.74 to 0.97)	20
	IACS	9	502	0.97 (0.67 to 1.28)	75	0.47 (0.32 to 0.70)	0
	IAHA	35	4782	1.16 (1.01 to 1.31)	87	0.82 (0.75 to 0.90)	23
	*Surgical*						
	Lavage	4	434	0.46 (0.24 to 0.68)	54	0.91 (0.60 to 1.37)	0
	Overall	208	40 243	1.09 (1.03 to 1.14)	90	0.75 (0.72 to 0.79)	42
Function	*Oral*						
	Glucosamine	20	2849	1.10 (0.88 to 1.31)	90	0.64 (0.49 to 0.82)	69
	Chondroitin	12	1924	0.98 (0.80 to 1.17)	81	0.63 (0.47 to 0.85)	61
	Glucosamine+chondroitin	5	1058	0.71 (0.32 to 1.10)	90	0.85 (0.71 to 1.02)	0
	Paracetamol	4	1373	0.73 (0.49 to 0.97)	83	0.92 (0.78 to 1.09)	0
	NSAIDs	44	14 613	1.02 (0.94 to 1.10)	88	0.64 (0.59 to 0.70)	50
	*Physical/topical*						
	PEMF	17	914	0.66 (0.47 to 0.84)	69	0.63 (0.47 to 0.84)	0
	Topical NSAIDs	17	3717	1.34 (1.10 to 1.58)	93	0.71 (0.58 to 0.87)	81
	*Needle/injection*						
	Acupuncture	12	2453	1.01 (0.78 to 1.23)	89	0.85 (0.72 to 1.00)	38
	IACS	6	421	0.62 (0.29 to 0.95)	80	0.68 (0.40 to 1.18)	0
	IAHA	25	3315	1.02 (0.87 to 1.18)	84	0.84 (0.74 to 0.96)	23
	*Surgical*						
	Lavage	4	425	0.47 (0.15 to 0.78)	77	0.93 (0.62 to 1.40)	0
	Overall	166	33 062	0.98 (0.93 to 1.04)	89	0.71 (0.67 to 0.75)	56
Stiffness	*Oral*						
	Glucosamine	9	1568	1.20 (0.77 to 1.63)	95	0.82 (0.63 to 1.05)	39
	Chondroitin	2	793	0.48 (0.12 to 0.83)	88	1.00 (0.77 to 1.30)	0
	Glucosamine+chondroitin	2	707	0.57 (−0.05 to 1.18)	92	0.91 (0.72 to 1.15)	0
	Paracetamol	3	644	0.63 (0.31 to 0.95)	83	0.95 (0.72 to 1.24)	0
	NSAIDs	29	8750	0.91 (0.84 to 0.99)	78	0.73 (0.67 to 0.80)	20
	*Physical/topical*						
	PEMF	6	378	0.49 (0.22 to 0.76)	66	0.71 (0.43 to 1.17)	0
	Topical NSAIDs	10	2578	1.01 (0.80 to 1.23)	90	0.93 (0.84 to 1.03)	0
	*Needle/injection*						
	Acupuncture	7	1181	0.93 (0.67 to 1.20)	80	0.79 (0.66 to 0.94)	0
	IACS	3	168	0.65 (0.22 to 1.08)	72	0.83 (0.34 to 2.04)	0
	IAHA	3	2462	0.83 (0.67 to 1.00)	82	0.88 (0.77 to 1.00)	0
	*Surgical*						
	Lavage	2	267	0.28 (0.04 to 0.53)	44	1.00 (0.46 to 2.16)	0
	Overall	91	19 490	0.83 (0.79 to 0.87)	87	0.83 (0.79 to 0.87)	1

IACS, intra-articular corticosteroid; IAHA, intra-articular hyaluronic acid; NSAIDs, non-steroidal anti-inflammatory drugs; PEMF, pulsed-electromagnetic field therapy.

### Determinants of PCE

#### Subgroup analysis

##### Sample size

Comparing the results from all trials, the PCE was higher in larger trials. In trials with >100 participants per arm, the PCE for pain reduction was 0.85 (95% CI 0.68 to 1.05) with chondroitin, 0.79 (95% CI 0.61 to 1.03) with glucosamine, 0.79 (95% CI 0.64 to 0.98) with glucosamine plus chondroitin, 0.85 (95% CI 0.70 to 1.03) with paracetamol, 0.72 (95% CI 0.66 to 0.78) with NSAIDs, 0.92 (95% CI 0.85 to 0.99) with topical NSAIDs, 0.91 (95% CI 0.82 to 1.01) with IAHA, and 0.98 (95% CI 0.86 to 1.12) with acupuncture (see online supplementary table S1).

##### Route of delivery

The PCE was lowest when the treatment was delivered by oral medication (PCE=0.70, 95% CI 0.66 to 0.75), higher when delivered via physical means, for example, PEMF (PCE=0.80, 95% CI 0.64 to 0.99) or by use of needles/injection (PCE=0.81, 95% CI 0.75 to 0.88), and highest with more invasive or surgical interventions, for example, joint lavage (PCE=0.91, 95% CI 0.60 to 1.37). In addition, the PCE with topical NSAIDs (PCE=0.85, 95% CI 0.77 to 0.93) was higher than its counterpart oral NSAIDs (PCE=0.70, 95% CI 0.70 to 0.65) (see online supplementary table S2).

##### Other determinants

The PCE was significantly higher in trials that applied allocation concealment (PCE=0.81, 95% CI 0.76 to 0.85) than in trials that did not (PCE=0.70, 95% CI 0.66 to 0.75), were conducted in developed countries and publicly funded, and with longer duration of treatment. However, the PCE was not significantly affected by the targeted joint, the chance of receiving the active treatment or the setting (primary care vs secondary care) in which the treatment was received (see online supplementary table S2).

#### Meta-regression

In meta-regression of PCE for pain, 467 observations were included. The PCE significantly increased in treatments involving needles or injection (β=0.119, 95% CI 0.056 to 0.182, p=0.000), physical therapy, for example, PEMF (β=0.208, 95% CI 0.029 to 0.387, p=0.023), and topical cream (β=0.174, 95% CI 0.104 to 0.244, p=0.000). It also significantly increased with longer duration of treatment (β=0.002, 95% CI 0.001 to 2.310, p=0.021), when sample size was larger than 100 per arm (β=0.177, 95% CI 0.121 to 0.232, p=0.000) and when trials were public funded (β=0.086, 95% CI 0.018 to 0.155, p=0.014). Other contextual factors such as baseline pain, mean age, percentage of women, chance of receiving active treatment and methodological aspects were not found to be significant determinants after adjustment for other factors (see online supplementary table S3).

## Discussion

This study focused on the overall treatment effect and the percentage of that effect that is attributable to contextual (placebo) effects (PCE). Using placebo-controlled RCT data in OA, we examined a sample of contrasting treatments including complementary medicines, nutraceuticals, oral drugs, topical NSAIDs, compounds administered through intra-articular injection and joint lavage. We found that the overall treatment effect of these 11 diverse treatments in reducing OA pain ranged from 0.46 SD (joint lavage) to 1.37 SD (topical NSAIDs), of which 91% of the improvement with lavage and 85% of the improvement with topical NSAIDs is explained by contextual effects. On average, the contextual effect contributed 75% to the overall treatment effect for the included treatments for pain in OA. We also found that PCE varied across treatments, ranging from the lowest with IACS (0.47, 95% CI 0.32 to 0.70) to the highest with joint lavage (0.91, 95% CI 0.60 to 1.37). Two factors known to influence the magnitude of placebo effect in OA RCTs, the mode of delivery and sample size of the study,^2^ also affected the magnitude of the PCE. The finding on sample size reaffirmed the ‘small study effect’ where smaller trials often report larger benefit of treatment over placebo, in which, as a ratio between ES of placebo and treatment, PCE would be smaller. This finding corresponds with the findings of our funnel plot. PCE also increased with longer duration of treatment and in trials with public funding source. Other factors such as percentage of women and methodological aspects did not affect the size of the PCE.

Although this is the first study in OA for PCE, the findings accord with those of SRs examining treatment of other chronic conditions. Kirsch and colleagues have reported that 75% of the overall drug effect of antidepressants can be explained by placebo effect.^21^ A more recent study using RCT data in all diseases found that the non-specific effects (contextual effects) account for roughly 60% of all treatment effects with variation between conditions.^22^

In the first RCT undertaken in 1945 examining the efficacy of streptomycin in tuberculosis, the treatment effect was defined as the difference between outcomes in the treatment and placebo groups.^23^ Since then, the separation of the treatment from the placebo group in terms of improved outcomes has been the main focus of interest in all RCTs. No published RCTs examined in this review have presented results as overall treatment effect and PCE, and even when the overall treatment effect was very large, all conclusions concerning efficacy were based solely on the difference between treatment and placebo groups. As far as we are aware, no other placebo-controlled RCTs in OA or other musculoskeletal conditions have used this method of expressing the results and we believe that this is also true in other disease areas.

There are several clinical implications for these findings. First, the overall treatment effects of the OA therapies examined are moderate to large with the majority being well above the clinically significant level of the current MCID (0.5 SD) suggested by the National Institute for Health and Care Excellence.^9^ This important observation is contrary to the general opinion that most treatments for OA are relatively weak, which encourages pessimism concerning treatment of people with OA.

Second, ordering these treatments according to strength of overall treatment ES gives a very different hierarchy to that obtained when ordered according to traditional standardised ES derived from the separation of treatment from placebo. This change in focus from specific to overall treatment ES more accurately reflects the real benefit that patients might expect from these treatments in clinical practice and better informs clinicians and patients about expected treatment outcomes.

Third, the majority of benefit obtained from treatments in OA derives from contextual rather than specific treatment effects, emphasising the potential importance and magnitude of contextual response in patient care. For example, while the overall effect of paracetamol is 0.65 (95% CI 0.46 to 0.83), 87% of this is attributable to contextual effects (PCE=0.87, 95% CI 0.73 to 1.03). This provides some explanation as to why paracetamol may have little benefit on its own—an ‘impure’ placebo.

Furthermore, presentation of overall treatment effect and PCE provides a solution for the ‘efficacy paradox’.^12^ Taking IAHA and IACS as examples, although the overall treatment effect of IAHA (1.16) was slightly higher than IACS (0.97), contextual effects explain much more of the effect of IAHA than they do for IACS (IAHA 82% vs IACS 47%) and this helps to explain why IAHA may be effective in the clinical setting but not in RCTs. Patient expectancy from IAHA may be enhanced by being told that hyaluronic acid is a relatively new treatment, that it is a natural product, that it has beneficial effects on the cartilage and joint tissues, and is very effective for OA pain, and this may result in a good postinjection outcome. In contrast, IACS is an old treatment and steroid is a considered a potent drug with recognised potential side effects, so proportionally more of its benefits in many clinical contexts may need to reside on its specific treatment effects.^24^

Therefore, when reporting RCTs, focusing on the overall treatment ES of a particular treatment is likely to provide a better idea of what to expect in routine care than emphasising the ES based solely on the difference between treatment and placebo. Furthermore, explaining to practitioners the proportion of a treatment effect that is explained by contextual effects emphasises the importance of context in daily clinical practice. Even the initial relief of severe pain from parenteral opioids, when the patient knows they are receiving it, predominantly results from contextual effects and expectancy.^25^ Practitioners may, therefore, be encouraged to optimise their patient–practitioner interaction and other contextual factors that are within their control.^24^

There are several limitations to this study. First, we did not examine all treatments for OA but selected a sample of treatments that varies in terms of mechanism of action and mode of delivery. Although we investigated a range of representatives OA treatments including non-pharmacological, pharmacological, surgical and complementary treatments, it is possible that our findings of a generally good high overall treatment ES and a high PCE will not be confirmed in other treatments for OA in placebo-controlled clinical trials. Furthermore, some important treatments for OA such as exercise, education, weight loss and arthroplasty are not readily amenable to a placebo or sham intervention, and in the absence of placebo-controlled randomised trials, it is very difficult to estimate the contribution of contextual effect to such interventions, just as it is difficult to estimate their traditional ES over placebo.

Second, the new hierarchy based on overall treatment effect shows topical NSAIDs, with its large PCE (85%) to be the best treatment for OA pain. However, it is hard to believe that topical NSAIDs are better than IACS in terms of overall treatment ES. It is possible that differences in patient selection may help to explain this apparent discrepancy. For example, patients selected for trials of IACS or surgical treatments may be more likely to be those experiencing unrelieved pain and may be more difficult to treat than patients with less resilient symptoms who more commonly may be selected for trials of topical NSAIDs and oral medications. This could have explained why participants in trials of topical NSAIDs show larger improvement from baseline than those in trials of IACS. However, we examined baseline pain and duration of disease/pain of patients in trials of different treatments and no apparent differences were found. This suggests that differences in other contextual factors, such as participant expectancy (largely influenced by the trial information received), treatment experience and participant–practitioner interaction, may be responsible for this result. However, we were unable to examine these patient-level contextual factors as they were rarely measured and reported in clinical trials, which is also the case for the PCE. Though proxy indicators such as chance of receiving active treatment and percentage of women in each trial were examined in meta-regression of PCE, they may be less sensitive to change. Thus, further study using individual patient data is warranted.

Since this is the first time that the overall treatment effect and PCE have been examined in OA, much of the interpretation of the hierarchy and proportions of specific and contextual effects has yet to be discovered. Further study is, therefore, warranted to better understand this. As the large overall treatment ES estimated in this study contains contextual effect, which include the regression to the mean or the Hawthorn effect, care must be taken when applying this measure. It should never be used alone but with the PCE.

Third, the contextual effect investigated in this study is a combination of placebo effect and other spontaneous effects, such as the Hawthorne effect, natural fluctuation in disease and regression to the mean. However, the PCE is not a measure that only counts the placebo group effect, but a measure that counts both the placebo and treatment group effects. In such a situation, other spontaneous effects such as regression to the mean and the Hawthorn effect (being observed) are subtracted in the log transform calculation. Such a simple estimate provides a clinically useful tool to estimate the PCE without further examination for other spontaneous effects, which are often not measurable from the trial data. Nevertheless, more research is warranted to investigate these components of the contextual effect and their roles in overall treatment effect.

Finally, we made no attempt to identify unpublished trials as do many other SRs. However, unpublished trials are more likely to have insignificant findings and a smaller separation between treatment and placebo, a higher PCE would be expected. Therefore, by not including unpublished studies the PCE found in our study is more likely to be a conservative estimate of the true PCE.

In conclusion, this study suggests that the overall treatment benefits from diverse treatments of OA are more than those reported in RCTs and predominantly result from the contextual effects rather than the specific effects of treatments. Reporting the overall treatment effect and the PCE together, in addition to traditional difference between treatment and placebo, helps to better translate RCT evidence into clinical practice, reduces the ‘efficacy paradox’ and emphasises to practitioners the importance of contextual factors in clinical care. More research is needed to identify key contextual factors that may work on their own in a generic fashion or which may show interactions with specific treatment effects.

## Supplementary Material

Web supplement

Web references
