# Supplementary material for: Examination of overall treatment effect and the proportion attributable to contextual effect in osteoarthritis: meta-analysis of randomised controlled trials
Source: Ann Rheum Dis. 2016 Feb 16;75(11):1964–70. doi: 10.1136/annrheumdis-2015-208387 (PMC5099197; doi:10.1136/annrheumdis-2015-208387)
Supplement: Web supplement [file annrheumdis-2015-208387-s1.pdf]

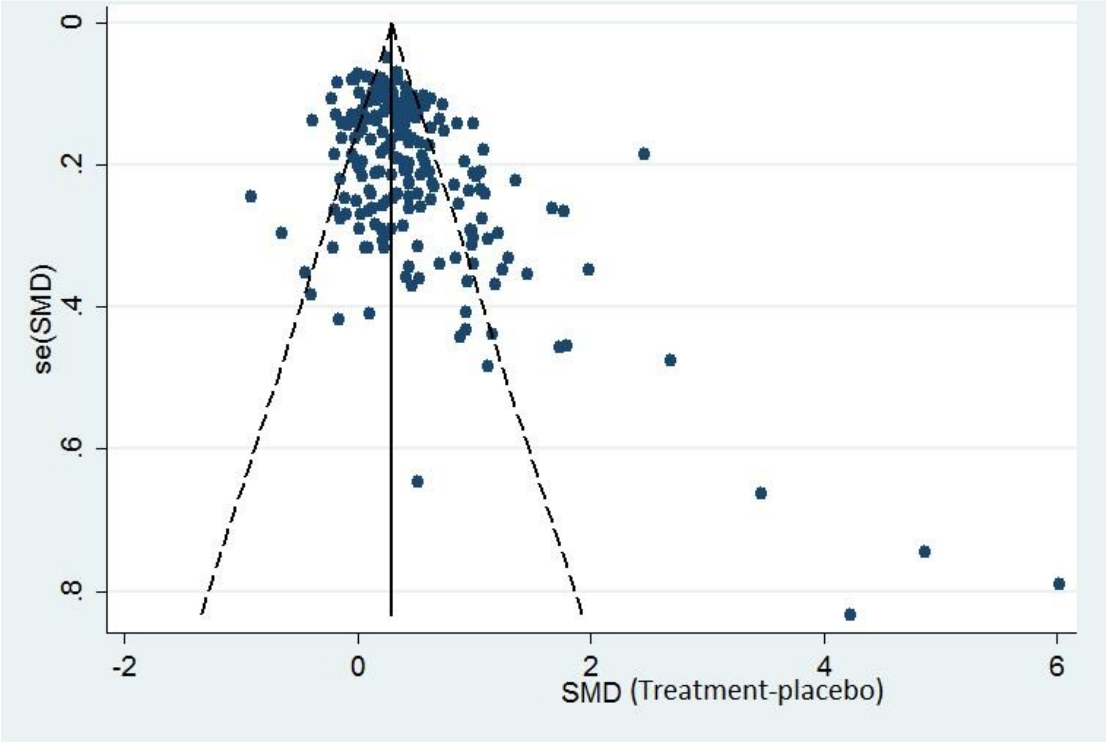

Online supplementary Figure S1. Funnel plot of traditional effect size between treatment and placebo groups (Egger's test p<0.001)

Online supplementary Table S1 Comparison between all trials and trials with sample size over 100 per arm for pain outcome

		PCE (95%Confidence interval)	
Outcome	Treatment	All trials	Trials with sample size≥100 per arm
Pain	**Oral**		
	Glucosamine	0.67(0.53, 0.84)	0.79 (0.61, 1.03)
	Chondroitin	0.68(0.55, 0.84)	0.85 (0.68, 1.05)
	NSAIDs	0.70(0.65, 0.75)	0.72 (0.66, 0.78)
	GS+CS	0.76(0.62, 0.93)	0.79 (0.64, 0.98)
	Paracetamol	0.87(0.73, 1.03)	0.85 (0.70, 1.03)
	**Topical**		
	PEMF	0.80(0.64, 0.99)	-
	Topical NSAIDs	0.85(0.77, 0.93)	0.92 (0.85, 0.99)
	**Needle/injection**		
	IACS	0.47(0.32, 0.70)	-
	IAHA	0.82(0.75, 0.90)	0.91 (0.82, 1.01)
	Acupuncture	0.85(0.74, 0.97)	0.98 (0.86, 1.12)
	**Surgical**		
	Lavage	0.91(0.60, 1.37)	-
	**Overall**	0.75 (0.72, 0.79)	0.81 (0.77, 0.85)
Function	**Oral**		
	NSAIDs	0.64(0.59, 0.70)	0.64 (0.58, 0.70)
	Chondroitin	0.63(0.47, 0.85)	0.69 (0.40, 1.17)
	Glucosamine	0.64(0.49, 0.82)	0.67 (0.45, 0.96)
	GS+CS	0.85(0.71, 1.02)	0.85 (0.70, 1.03)
	Paracetamol	0.92(0.78, 1.09)	0.90 (0.73, 1.11)
	**Topical**		
	PEMF	0.63(0.47, 0.84)	-
	Topical NSAIDs	0.71(0.58, 0.87)	0.78 (0.60, 1.01)
	**Needle/injection**		
	IACS	0.68(0.40, 1.18)	-
	IAHA	0.84(0.74, 0.96)	0.95 (0.83, 1.08)
	Acupuncture	0.85(0.72, 1.00)	0.95 (0.83, 1.09)
	**Surgical**		
	Lavage	0.93(0.62, 1.40)	-
	**Overall**	0.71(0.67, 0.75)	0.73 (0.68, 0.79)
Stiffness	**Oral**		
	NSAIDs	0.73(0.67, 0.80)	0.75 (0.69, 0.81)
	Glucosamine	0.82(0.63, 1.05)	0.83 (0.62, 1.12)
	GS+CS	0.91(0.72, 1.15)	0.91 (0.72, 1.15)
	Paracetamol	0.95(0.72, 1.24)	0.94 (0.72, 1.24)
	Chondroitin	1.00(0.77, 1.30)	1.00 (0.77, 1.30)

**Topical**		
PEMF	0.71(0.43, 1.17)	
Topical NSAIDs	0.93(0.84, 1.03)	0.95 (0.86, 1.05)
**Needle/injection**		
Acupuncture	0.79(0.66, 0.94)	0.82 (0.64, 1.04)
IACS	0.83(0.34, 2.04)	-
IAHA	0.88(0.77, 1.00)	0.88 (0.73, 1.06)
**Surgical**		
Lavage	1.00(0.46, 2.16)	-
**Overall**	0.83(0.79, 0.87)	0.83 (0.79, 0.88)

PCE: proportion attributable to contextual effect; GS: glucosamine sulphate, CS: chondroitin sulphate, IACS: intra-articular corticosteroid, IAHA: intra-articular hyaluronic acid, PEMF: pulsed-electromagnetic field therapy, NSAIDs: non-steroidal anti-inflammatory drugs

Online supplementary Table S2 Subgroup analyses of the proportion attributable to contextual effect in osteoarthritis

Outcome	Subgroups	No. of trials	No. of patients	PCE (95%CI)	I^2%
**Pain**	**Treatment administration**				
	Oral medication	105	26380	0.70(0.66, 0.75)	54
	PEMF	18	1005	0.80(0.64, 0.99)	0
	Needle/injection	61	8031	0.81(0.75, 0.88)	24
	Topical	20	4405	0.85(0.77, 0.93)	35
	Surgery	4	434	0.91(0.60, 1.37)	0
	**Duration of treatment**				
	≤4 weeks	114	17775	0.74(0.69, 0.79)	35
	4-8 weeks	76	12807	0.76(0.71, 0.83)	38
	9-13 weeks	90	20258	0.80(0.76, 0.84)	23
	>13 weeks	52	11221	0.88(0.84, 0.93)	0
	**Chance of receiving active treatment**				
	33%	7	1385	0.92(0.78, 1.07)	0
	50%	110	14649	0.75(0.70, 0.81)	40
	60%	2	624	0.83(0.69, 0.98)	0
	67%	60	13925	0.71(0.65, 0.77)	53
	75%	19	6141	0.79(0.74, 0.85)	15
	80%	8	3262	0.75(0.63, 0.90)	63
	83%	2	264	0.68(0.33, 1.39)	77
	**Adequate allocation concealment**				
	No	113	21292	0.70(0.66, 0.75)	47
	Yes	95	18951	0.81(0.76, 0.85)	32
	**Blinding of participants**				
	No	15	940	0.62(0.48, 0.79)	37
	Yes	193	39298	0.76(0.73, 0.79)	42
	**Setting**				
	Secondary care	184	34018	0.74(0.71, 0.78)	40
	Primary care	17	4092	0.79(0.67, 0.92)	59
	**Funding source**				
	Public	50	7942	0.86 (0.79, 0.92)	9
	Industry	113	27782	0.77 (0.73, 0.81)	38
	Unclear	45	4525	0.60 (0.52, 0.71)	57
	**Joint site**				
	Spine	5	379	0.63 (0.44, 0.89)	0

	Hip	11	2228	0.66 (0.51, 0.85)	26
	Knee	147	29294	0.78 (0.74, 0.82)	42
	Hand	5	1036	0.82 (0.67, 1.01)	22
	Foot	3	243	0.96 (0.67, 1.38)	0
	TMJ	2	93	0.80 (0.16, 3.97)	0
	Mixed	35	6977	0.64 (0.57, 0.72)	43
	**Country**				
	Developing	11	950	0.49 (0.33, 0.72)	80
	Mixed	1	329	0.72 (0.53, 0.98)	/
	Developed	196	38971	0.77 (0.74, 0.81)	33
**Function**	**Treatment administration**				
	Oral	85	21811	0.67 (0.62, 0.73)	60
	PEMF	17	914	0.63 (0.47, 0.84)	0
	Topical	17	3717	0.71 (0.58, 0.87)	81
	Needle/injection	43	6189	0.84 (0.77, 0.93)	20
	Surgery	4	425	0.93 (0.62, 1.40)	0
	**Duration of treatment**				
	≤4 weeks	80	13402	0.65(0.58, 0.72)	54
	4-8 weeks	56	9929	0.75(0.68, 0.83)	36
	9-13 weeks	69	16564	0.74(0.68, 0.81)	52
	>13 weeks	47	4683	0.88(0.83, 0.95)	0
	**Chance of receiving active treatment**				
	33%	6	1244	0.82 (0.69, 0.98)	0
	50%	92	12530	0.74 (0.67, 0.80)	45
	60%	2	621	0.46 (0.20, 1.06)	95
	67%	42	9713	0.68 (0.60, 0.76)	61
	75%	15	5440	0.72 (0.66, 0.78)	0
	80%	8	3311	0.70 (0.58, 0.86)	56
	83%	1	192	0.49 (0.29, 0.82)	/
	**Adequate allocation concealment**				
	No	85	17187	0.67 (0.62, 0.73)	54
	Yes	81	15863	0.79 (0.73, 0.85)	40
	**Blinding of participants**				
	No	11	732	0.58 (0.36, 0.92)	61
	Yes	155	32318	0.73 (0.69, 0.78)	49
	**Setting**				
	Secondary care	148	28862	0.70 (0.65, 0.74)	54
	Primary care	18	4200	0.78 (0.67, 0.92)	59
	**Funding source**				

	Public	42	7378	0.85 (0.78, 0.93)	15
	Industry	93	23271	0.68 (0.63, 0.73)	63
	Unclear	31	2420	0.63 (0.51, 0.78)	49
	**Joint site**				
	Spine	3	232	0.42 (0.19, 0.92)	72
	Hip	9	2091	0.54 (0.36, 0.81)	58
	Knee	124	25328	0.74 (0.69, 0.79)	55
	Hand	3	936	0.70 (0.55, 0.89)	0
	Foot	2	45	0.79 (0.35, 1.80)	0
	TMJ	2	93	0.84 (0.27, 2.65)	0
	Mixed	23	4339	0.59 (0.49, 0.70)	56
	**Country**				
	Developing	10	1000	0.86 (0.74, 1.01)	0
	Mixed	1	329	0.77 (0.54, 1.10)	/
	Developed	155	31728	0.72 (0.68, 0.76)	53
**Stiffness**	**Treatment administration**				
	Oral	45	12468	0.77 (0.71, 0.83)	25
	PEMF	6	378	0.71 (0.43, 1.17)	0
	Topical	10	2572	0.93 (0.84, 1.03)	0
	Needle/injection	28	3811	0.84 (0.76, 0.94)	0
	Surgery	2	267	1.00 (0.46, 2.16)	0
	**Duration of treatment**				
	≤4 weeks	36	5430	0.80(0.72, 0.88)	0
	4-8 weeks	29	4729	0.71(0.60, 0.84)	47
	9-13 weeks	36	9507	0.82(0.77, 0.88)	0
	>13 weeks	23	6156	0.90(0.83, 0.97)	0
	**Chance of receiving active treatment**				
	33%	3	482	0.73 (0.51, 1.05)	20
	50%	50	7482	0.86 (0.81, 0.93)	0
	60%	2	618	0.76 (0.61, 0.95)	0
	67%	26	6307	0.76 (0.68, 0.85)	27
	75%	7	2706	0.76 (0.66, 0.88)	1
	80%	3	1896	0.94 (0.81, 1.08)	0
	**Adequate allocation concealment**				
	No	47	8992	0.79 (0.74, 0.85)	0
	Yes	44	10493	0.85 (0.79, 0.91)	16
	**Blinding of participants**				
	No	8	413	0.64 (0.42, 0.97)	0
	Yes	83	19083	0.83 (0.79, 0.87)	1

**Setting**				
Secondary care	80	16864	0.81 (0.77, 0.86)	0
Primary care	11	2621	0.88 (0.80, 0.98)	0
**Funding source**				
Public	26	5045	0.87 (0.79, 0.95)	0
Industry	53	13672	0.81 (0.76, 0.87)	13
Unclear	12	774	0.65 (0.51, 0.83)	4
**Joint site**				
Spine	/			
Hip	8	1953	0.71 (0.61, 0.83)	0
Knee	67	14494	0.85 (0.80, 0.90)	5
Hand	4	986	0.80 (0.63, 1.00)	0
Foot	1	28	1.00 (0.52, 1.91)	/
TMJ	/			
Mixed	11	2036	0.75 (0.64, 0.89)	13
**Country**				
Developing	3	393	0.94 (0.72, 1.24)	0
Mixed	1	329	0.82 (0.59, 1.14)	/
Developed	87	18774	0.82 (0.78, 0.86)	3

PCE= Proportional attributable to contextual effect; PEMF=Pulsed electromagnetic field therapy

Online supplementary Table S3 Meta-regression analysis of the determinants of the proportion attributable to contextual effects for pain in osteoarthritis

	β	(95%	CI)	p value
**Administration of treatment**				
Needle/injection vs. oral medication	0.119	(0.056	0.182)	0.000
PEMF vs. oral medication	0.208	(0.029	0.387)	0.023
Surgery vs. oral medication	0.249	(-0.016	0.514)	0.066
Topical vs. oral medication	0.174	(0.104	0.244)	0.000
**Baseline pain (1-100)**	0.002	(-0.001	0.004)	0.150
**Mean age, years**	0.000	(-0.007	0.007)	0.937
**Percentage of women, %**	-0.002	(-0.004	0.000)	0.109
**Duration of treatment, weeks**	0.002	(0.000	0.004)	0.021
**Chance of receiving active treatment, %**	0.162	(-0.067	0.391)	0.165
**Sample size≥100/arm (yes vs. no)**	0.177	(0.121	0.232)	0.000
**Adequate allocation concealment (yes vs. no)**	0.006	(-0.038	0.049)	0.802
**Adequate blinding of patients (yes vs. no)**	0.122	(-0.027	0.271)	0.108
**Funding source**				
Industry vs. public	-0.086	(-0.155	-0.018)	0.014
Unclear vs. public	-0.066	(-0.166	0.035)	0.199
**Cons**	-0.558	(-1.076	-0.039)	0.035

PEMF= Pulsed electromagnetic field therapy

Online supplementary - Search strategy in Medline (OVID)

The search terms of “osteoarthritis”, “randomised controlled trials” and each “treatment” were combined with “and” in literature search.

For osteoarthritis

1 osteoarthritis.mp. or exp osteoarthritis/
2 arthrosis.mp.
3 osteoarthr$.mp.
4 (degenerative adj2 arthritis).mp.
5 gonarthrosis.mp.
6 coxarthrosis.mp.
7 1or 2 or 3 or 4 or 5 or 6

For randomised controlled trial

1 randomized controlled trial.pt.
2 controlled clinical trial.pt.
3 randomized.ab.
4 placebo.ab.
5 clinical trials as topic.sh.
6 randomly.ab.
7 trial.ti.
8 1 or 2 or 3 or 4 or 5 or 6 or 7
9 (animals not (humans and animals)).sh.
10 8 not 9

For treatments

Acupuncture

1 exp Acupuncture Therapy/
2 acupunc$.tw.
3 electroacupunc$.tw.
4 meridian$.tw.
5 acupoints$.tw.
6 1 or 2 or 3 or 4 or 5

Pulsed electro-magnetic field therapy

1 Electromagnetic Field/
2 electromagnetic$.tw.
3 exp Electric Stimulation Therapy/
4 (electric$ adj3 stimulat$).tw.
5 (short$ adj3 wave&).tw.
6 or/1-5

Intra-articular injection of hyaluronic acid

1 viscosupplementation.mp. or exp Viscosupplementation/
2 hyaluronic acid.mp. or exp Hyaluronic Acid/
3 hyaluronan.mp.
4 hyaluronate.mp.
5 Hyalgan.mp.
6 Synvisc.mp.
7 Orthovisc.mp.
8 Artzal.mp.
9 Supartz.mp.
10 Suplasyn.mp.
11 BioHy.mp.
12 Euflexxa.mp.
13 Nuflexxa.mp.
14 Hylan GF-20.mp.
15 healonid.mp.
16 hylartil.mp.
17 hylartil.mp.
18 nrd 101.mp.
19 adant.mp.
20 or/1-20

Intra-articular injection of corticosteroid

1 exp Triamcinolone Acetonide/ or exp Triamcinolone/ or triamcinolone.mp.
2 exp Betamethasone/ or betamethasone.mp.
3 corticosteroid.mp. or exp Adrenal Cortex Hormones/
4 glucocorticoid.mp. or exp Glucocorticoids/
5 hydroxycorticosteroid.mp. or exp Hydroxycorticosteroids/
6 corticoid.mp.
7 exp Ketosteroids/ or ketosteroid.mp.
8 androstenedione.mp. or exp Androstenedione/
9 cortivazol.mp.
10 exp Methylprednisolone/ or methylprenisolone.mp.
11 injections, intra-articular.mp. or exp Injections, Intra-Articular/
12 intraarticular inject$.tw.
13 intra-articular inject$.tw.
14 or/1-10

Paracetamol

1 exp Acetaminophen/
2 ("Acetaminophen" or "paracetamol").mp.
3 1 or 2

Non-steroid anti-inflammatory drug

1 exp Anti-Inflammatory Agents, Non-Steroidal/
2 NSAIDs.mp.
3 cyclooxygenase.mp.
4 cox* inhibitor.mp.
5 *coxib/
6 Lodine.mp.
7 celecoxib.mp.
8 Celebrex.mp.
9 rofecoxib.mp.
10 Vioxx.mp.
11 meloxicam.mp.
12 Mobic.mp.
13 *Naprosyn/
14 Anaprox*.mp.
15 Naprapac Aleve.mp.
16 (Cataflam or Voltaren or Arthrotec or Pennsaid).mp.
17 lumiracoxib.mp.
18 etoricoxib.mp.
19 Motrin.mp.
20 Profen.mp.
21 Vicoprofen.mp.
22 Combunox.mp.
23 Advil.mp.
24 Dolobid.mp.
25 Nalfon.mp.
26 Ansaid.mp.
27 indometacin.mp.
28 Indocin.mp.
29 Indo-Lemmon.mp.
30 Indomethagan.mp.
31 Oruvail.mp.
32 Toradol.mp.
33 Mefenamic Acid.mp. or exp Mefenamic Acid/
34 Ponstel.mp.
35 Nabumetone.mp.
36 Relafen.mp.
37 Oxaprozin.mp.
38 Daypro.mp.
39 Piroxicam.mp. or exp Piroxicam/
40 Feldene.mp.
41 Sulindac.mp. or exp Sulindac/
42 Clinoril.mp.
43 Tolmetin.mp. or exp Tolmetin/

44 Tolectin.mp.
45 Valdecoxib.mp.
46 Bextra.mp.
47 Diacerein.mp.
48 Diacerhein.mp.
49 Rhein.mp.
50 Anthraquinones.mp. or exp Anthraquinones/
51 Diacetyilrhein.mp.
52 ART 50.mp.
53 Cyclooxygenase Inhibitors.mp. or exp Cyclooxygenase Inhibitors/
54 exp Cyclooxygenase Inhibitors/ or exp Cyclooxygenase 2 Inhibitors/
55 exp Aspirin/ or Aspirin.mp.
56 Etodolac.mp. or exp Etodolac/
57 naproxen.mp. or exp Naproxen/
58 Diclofenac.mp. or exp Diclofenac/
59 Ibuprofen.mp. or exp Ibuprofen/
60 Diflunisal.mp. or exp Diflunisal/
61 Fenoprofen.mp. or exp Fenoprofen/
62 Flurbiprofen.mp. or exp Flurbiprofen/
63 Indomethacin.mp. or exp Indomethacin/
64 Ketoprofen.mp. or exp Ketoprofen/
65 exp Ketorolac/ or Ketorolac.mp.
66 or/1-65

Joint lavage
1 (arthroscop$ adj40 debridement).tw.
2 (arthroscop$ adj40 irrigation).tw.
3 (arthroscop$ adj40 lavage).tw.
4 (arthroscop$ adj40 washout).tw.
5 exp arthroscopy/ and (exp debridement/ or exp therapeutic Irrigation/)
6 (needle$ adj40 debridement).tw.
7 (needle$ adj40 irrigation).tw.
8 (needle$ adj40 lavage).tw.
9 (needle$ adj40 washout).tw.
10 1 or 2 or 3 or 4 or 5 or 6 or 7 or 8 or 9

Glucosamine
1. exp glucosamine/
2. glucosamine.rn, tw.
3. acetylglucosamine.tw, rn.
4. n-acetylglucosamine.tw.
5. n-acetyl-d-glucosamine.tw.
6. or/1-6

Chondroitin
1. exp CHONDROITIN/
2. chondroitin.sh,rn,tw.
3. 1 or 2
