# Supplementary material for: Examination of overall treatment effect and the proportion attributable to contextual effect in osteoarthritis: meta-analysis of randomised controlled trials
Source: Ann Rheum Dis. 2016 Feb 16;75(11):1964–70. doi: 10.1136/annrheumdis-2015-208387 (PMC5099197; doi:10.1136/annrheumdis-2015-208387)
Supplement: Web references [file annrheumdis-2015-208387-s2.pdf]

Supplementary File

References of included studies

Included trials of acupuncture [1-17]

Included trials of chondroitin [18-30]

Included trials of glucosamine [18, 30-49]

Included trials of glucosamine plus chondroitin [18, 50-53]

Included trials of intra-articular injection of corticosteroid [54-63]

Included trials of intra-articular injection of hyaluronic acid [61, 64-99]

Included trials of joint lavage [62, 98-100]

Included trials of non-steroidal anti-inflammatory drugs [18, 45, 64, 101-156]

Included trials of paracetamol [36, 108, 136, 157-159]

Included trials of pulsed electromagnetic field therapy [160-177]

Included trials of topical non-steroidal anti-inflammatory drugs [139, 140, 148, 178-194]

1. Berman BM, Lao LX, Langenberg P, Lee WL, Gilpin AMK, Hochberg MC. Effectiveness of acupuncture as adjunctive therapy in osteoarthritis of the knee - A randomized, controlled trial. *Annals of Internal Medicine* 2004; 141(12): 901-10.

2. Foster NE, Thomas E, Barlas P, et al. Acupuncture as an adjunct to exercise based physiotherapy for osteoarthritis of the knee: Randomised controlled trial. *British Medical Journal* 2007; 335(7617): 436-40.

3. Huang W, Bliwise DL, Carnevale CV, Kutner NG. Acupuncture for pain and sleep in knee osteoarthritis. *Journal of the American Geriatrics Society* 2010; 58(6): 1218-20.

4. Jubb R, Tukmachi E, Jones P, Dempsey E, Waterhouse L, Brailsford S. A blinded randomised trial of acupuncture (manual and electroacupuncture) compared with a non-penetrating sham for the symptoms of osteoarthritis of the knee. *Acupuncture in Medicine* 2008; 26(2): 69-78.

5. Lu T, Wei I, Liu Y, et al. Immediate effects of acupuncture on gait patterns in patients with knee osteoarthritis. *Chinese medical journal* 2010; 123(2): 165-72.

6. Lundeberg T, Eriksson SV, Lundeberg S, Thomas M. Effect of acupuncture and naloxone in patients with osteoarthritis pain. A sham acupuncture controlled study. *Pain Clinic* 1991; 4(3): 155-61.

7. Mavrommatis C, Argyra E, Vadalouka A, Vasilakos D. Acupuncture as an adjunctive therapy to pharmacological treatment in patients with chronic pain due to osteoarthritis of the knee: A 3-armed, randomized, placebo-controlled trial. *Pain* 2012; 153(8): 1720-6.

8. Miller E, Maimon Y, Rosenblatt Y, et al. Delayed effect of acupuncture treatment in OA of the knee: A blinded, randomized, controlled trial. *Evidence-based Complementary and Alternative Medicine* 2011; 2011(792975).

9. Sangdee C, Teekachunhatean S, Sananpanich K, et al. Electroacupuncture versus diclofenac in symptomatic treatment of osteoarthritis of the knee: a randomized controlled trial. *BMC complementary and alternative medicine* 2002; 2: 3.

10. Scharf H, Mansmann U, Streitberger K, et al. Acupuncture and knee osteoarthritis: a three-armed randomized trial. *Annals of Internal Medicine* 2006; 145(1): 12.

11. Suarez-Almazor ME, Looney C, Liu Y, et al. A randomized controlled trial of acupuncture for osteoarthritis of the knee: Effects of patient-provider communication. *Arthritis Care and Research* 2010; 62(9): 1229-36.

12. Takeda W, Wessel J. Acupuncture for the treatment of pain of osteoarthritic knees. *Arthritis Care and Research* 1994; 7(3): 118-22.

13. Thomas M, Eriksson SV, Lundeberg T. A comparative study of diazepam and acupuncture in patients with osteoarthritis pain: a placebo controlled study. *The American journal of Chinese medicine* 1991; 19(2): 95-100.

14. Vas J, Mendez C, Perea-Milla E, et al. Acupuncture as a complementary therapy to the pharmacological treatment of osteoarthritis of the knee: randomised controlled trial. *British Medical Journal* 2004; 329(7476): 1216-9.

15. Weiner DK, Moore CG, Morone NE, Lee ES, Kent Kwoh C. Efficacy of periosteal stimulation for chronic pain associated with advanced knee osteoarthritis: a randomized, controlled clinical trial. *Clinical therapeutics* 2013; 35(11): 1703-20 e5.

16. White P, Prescott P, Lewith G. Does needling sensation (de qi) affect treatment outcome in pain? Analysis of data from a larger single-blind, randomised controlled trial. *Acupuncture in Medicine* 2010; 28(3): 120-5.

17. Witt C, Brinkhaus B, Jena S, et al. Acupuncture in patients with osteoarthritis of the knee: A randomised trial. *Lancet* 2005; 366(9480): 136-43.

18. Clegg DO, Reda DJ, Harris CL, et al. Glucosamine, chondroitin sulfate, and the two in combination for painful knee osteoarthritis. *New England Journal of Medicine* 2006; 354(8): 795-808.

19. Gabay C, Medinger-Sadowski C, Gascon D, Kolo F, Finckh A. Symptomatic effects of chondroitin 4 and chondroitin 6 sulfate on hand osteoarthritis: a randomized, double-blind, placebo-controlled clinical trial at a single center. *Arthritis and rheumatism* 2011; 63(11): 3383-91.

20. Kahan A, Uebelhart D, De Vathaire F, Delmas PD, Reginster JY. Long-term effects of chondroitins 4 and 6 sulfate on knee osteoarthritis: the study on osteoarthritis progression prevention, a two-year, randomized, double-blind, placebo-controlled trial. *Arthritis and rheumatism* 2009; 60(2): 524-33.

21. Bucsi L, Poor G. Efficacy and tolerability of oral chondroitin sulfate as a symptomatic slow-acting drug for osteoarthritis (SYSADOA) in the treatment of knee osteoarthritis. *Osteoarthritis and cartilage / OARS, Osteoarthritis Research Society* 1998; 6 Suppl A: 31-6.

22. Mazieres B, Combe B, Phan Van A, Tondut J, Grynfeltt M. Chondroitin sulfate in osteoarthritis of the knee: a prospective, double blind, placebo controlled multicenter clinical study. *The Journal of rheumatology* 2001; 28(1): 173-81.

23. Mazieres B, Hucher M, Zaim M, Garnero P. Effect of chondroitin sulphate in symptomatic knee osteoarthritis: a multicentre, randomised, double-blind, placebo-controlled study. *Annals of the rheumatic diseases* 2007; 66(5): 639-45.

24. Moller I, Perez M, Monfort J, et al. Effectiveness of chondroitin sulphate in patients with concomitant knee osteoarthritis and psoriasis: a randomized, double-blind, placebo-controlled study. *Osteoarthritis and cartilage / OARS, Osteoarthritis Research Society* 2010; 18 Suppl 1: S32-40.

25. Bourgeois P, Chales G, Dehais J, Delcambre B, Kuntz JL, Rozenberg S. Efficacy and tolerability of chondroitin sulfate 1200 mg/day vs chondroitin sulfate 3 x 400 mg/day vs placebo. *Osteoarthritis and cartilage / OARS, Osteoarthritis Research Society* 1998; 6 Suppl A: 25-30.

26. Railhac JJ, Zaim M, Saurel AS, Vial J, Fournie B. Effect of 12 months treatment with chondroitin sulfate on cartilage volume in knee osteoarthritis patients: a randomized, double-blind, placebo-controlled pilot study using MRI. *Clinical rheumatology* 2012; 31(9): 1347-57.

27. Uebelhart D, Malaise M, Marcolongo R, et al. Intermittent treatment of knee osteoarthritis with oral chondroitin sulfate: a one-year, randomized, double-blind, multicenter study versus placebo. *Osteoarthritis and cartilage / OARS, Osteoarthritis Research Society* 2004; 12(4): 269-76.

28. Uebelhart D, Thonar EJ, Delmas PD, Chantraine A, Vignon E. Effects of oral chondroitin sulfate on the progression of knee osteoarthritis: a pilot study. *Osteoarthritis and cartilage / OARS, Osteoarthritis Research Society* 1998; 6 Suppl A: 39-46.

29. Wildi LM, Raynauld JP, Martel-Pelletier J, et al. Chondroitin sulphate reduces both cartilage volume loss and bone marrow lesions in knee osteoarthritis patients starting as early as 6 months after initiation of therapy: a randomised, double-blind, placebo-controlled pilot study using MRI. *Annals of the rheumatic diseases* 2011; 70(6): 982-9.

30. Zegels B, Crozes P, Uebelhart D, Bruyere O, Reginster JY. Equivalence of a single dose (1200 mg) compared to a three-time a day dose (400 mg) of chondroitin 4&6 sulfate in patients with knee osteoarthritis. Results of a randomized double blind placebo controlled study. *Osteoarthritis and cartilage / OARS, Osteoarthritis Research Society* 2013; 21(1): 22-7.

31. D'Ambrosio E, Casa B, Bompani R, Scali G, Scali M. Glucosamine sulphate: a controlled clinical investigation in arthrosis. *Pharmatherapeutica* 1981; 2(8): 504-8.

32. Braham R, Dawson B, Goodman C. The effect of glucosamine supplementation on people experiencing regular knee pain. *British journal of sports medicine* 2003; 37(1): 45-9; discussion 9.

33. Cahlin BJ, Dahlstrom L. No effect of glucosamine sulfate on osteoarthritis in the temporomandibular joints--a randomized, controlled, short-term study. *Oral surgery, oral medicine, oral pathology, oral radiology, and endodontics* 2011; 112(6): 760-6.

34. Drovanti A, Bignamini AA, Rovati AL. Therapeutic activity of oral glucosamine sulfate in osteoarthrosis: a placebo-controlled double-blind investigation. *Clinical therapeutics* 1980; 3(4): 260-72.

35. Giordano N, Fioravanti A, Papakostas P, Montella A, Giorgi G, Nuti R. The efficacy and tolerability of glucosamine sulfate in the treatment of knee osteoarthritis: A randomized, double-blind, placebo-controlled trial. *Current therapeutic research, clinical and experimental* 2009; 70(3): 185-96.

36. Herrero-Beaumont G, Ivorra JAR, Trabado MDC, et al. Glucosamine sulfate in the treatment of knee osteoarthritis symptoms - A randomized, double-blind, placebo-controlled study using acetaminophen as a side comparator. *Arthritis and rheumatism* 2007; 56(2): 555-67.

37. Houpt JB, McMillan R, Wein C, Paget-Dellio SD. Effect of glucosamine hydrochloride in the treatment of pain of osteoarthritis of the knee. *The Journal of rheumatology* 1999; 26(11): 2423-30.

38. Hughes R, Carr A. A randomized, double-blind, placebo-controlled trial of glucosamine sulphate as an analgesic in osteoarthritis of the knee. *Rheumatology (Oxford, England)* 2002; 41(3): 279-84.

39. Frestedt JL, Walsh M, Kuskowski MA, Zenk JL. A natural mineral supplement provides relief from knee osteoarthritis symptoms: a randomized controlled pilot trial. *Nutrition journal* 2008; 7: 9.

40. Kawasaki T, Kurosawa H, Ikeda H, et al. Additive effects of glucosamine or risedronate for the treatment of osteoarthritis of the knee combined with home exercise: a prospective randomized 18-month trial. *Journal of bone and mineral metabolism* 2008; 26(3): 279-87.

41. Kwoh CK, Roemer FW, Hannon MJ, et al. Effect of oral glucosamine on joint structure in individuals with chronic knee pain: a randomized, placebo-controlled clinical trial. *Arthritis & rheumatology* 2014; 66(4): 930-9.

42. McAlindon T, Formica M, LaValley M, Lehmer M, Kabbara K. Effectiveness of glucosamine for symptoms of knee osteoarthritis: results from an internet-based randomized double-blind controlled trial. *The American journal of medicine* 2004; 117(9): 643-9.

43. Noack W, Fischer M, Forster KK, Rovati LC, Setnikar I. Glucosamine sulfate in osteoarthritis of the knee. *Osteoarthritis and cartilage / OARS, Osteoarthritis Research Society* 1994; 2(1): 51-9.

44. Pavelka K, Gatterova J, Olejarova M, Machacek S, Giacovelli G, Rovati LC. Glucosamine sulfate use and delay of progression of knee osteoarthritis: a 3-year, randomized, placebo-controlled, double-blind study. *Archives of internal medicine* 2002; 162(18): 2113-23.

45. Petersen SG, Beyer N, Hansen M, et al. Nonsteroidal anti-inflammatory drug or glucosamine reduced pain and improved muscle strength with resistance training in a randomized controlled trial of knee osteoarthritis patients. *Archives of physical medicine and rehabilitation* 2011; 92(8): 1185-93.

46. Pujalte JM, Llavore EP, Ylescupidez FR. Double-blind clinical evaluation of oral glucosamine sulphate in the basic treatment of osteoarthrosis. *Current medical research and opinion* 1980; 7(2): 110-14.

47. Rindone JP, Hiller D, Collacott E, Nordhaugen N, Arriola G. Randomized, controlled trial of glucosamine for treating osteoarthritis of the knee. *The Western journal of medicine* 2000; 172(2): 91-4.

48. Rozendaal RM, Koes BW, van Osch GJ, et al. Effect of glucosamine sulfate on hip osteoarthritis: a randomized trial. *Annals of internal medicine* 2008; 148(4): 268-77.

49. Zenk JL, Helmer TR, Kuskowski MA. The effects of milk protein concentrate on the symptoms of osteoarthritis in adults: an exploratory, randomized, double-blind, placebo-controlled trial. *Current Therapeutic Research* 2002; 63(7): 430-42.

50. Kanzaki N, Saito K, Maeda A, et al. Effect of a dietary supplement containing glucosamine hydrochloride, chondroitin sulfate and quercetin glycosides on symptomatic knee osteoarthritis: a randomized, double-blind, placebo-controlled study. *Journal of the science of food and agriculture* 2012; 92(4): 862-9.

51. Massari F, De Carvalho VA, Da Silva CR. Efficacy and safety of the association of glucosamine sulfate and chondroitin sulfate in the treatment of symptomatic knee osteoarthritis. [Portuguese]

Eficacia e seguranca da associacao de sulfato de glucosamina e sulfato de condroitina no tratamento sintomatico da osteoartrite de joelho. *Revista Brasileira de Medicina* 2014; 71(6-7).

52. Messier SP, Mihalko S, Loeser RF, et al. Glucosamine/chondroitin combined with exercise for the treatment of knee osteoarthritis: a preliminary study. *Osteoarthritis and cartilage / OARS, Osteoarthritis Research Society* 2007; 15(11): 1256-66.

53. Nguyen P, Mohamed SE, Gardiner D, Salinas T. A randomized double-blind clinical trial of the effect of chondroitin sulfate and glucosamine hydrochloride on temporomandibular joint disorders: a pilot study. *Cranio : the journal of craniomandibular practice* 2001; 19(2): 130-9.

54. Chao J, Wu C, Sun B, et al. Inflammatory characteristics on ultrasound predict poorer longterm response to intraarticular corticosteroid injections in knee osteoarthritis. *J Rheumatol* 2010; 37(3): 650-5.

55. Dieppe PA, Sathapatayavongs B, Jones HE, Bacon PA, Ring EF. Intra-articular steroids in osteoarthritis. *Rheumatol Rehabil* 1980; 19(4): 212-7.

56. Gaffney K, Ledingham J, Perry JD. Intra-articular triamcinolone hexacetonide in knee osteoarthritis: factors influencing the clinical response. *Ann Rheum Dis* 1995; 54(5): 379-81.

57. Hall M, Doherty S, Courtney P, Latief K, Zhang W, Doherty M. Ultrasound detected synovial change and pain response following intra-articular injection of corticosteroid and a placebo in symptomatic osteoarthritic knees: A pilot study. *Ann Rheum Dis*, 2014. http://onlinelibrary.wiley.com/o/cochrane/clcentral/articles/008/CN-00995008/frame.html (accessed.

58. Jones A, Doherty M. Intra-articular corticosteroids are effective in osteoarthritis but there are no clinical predictors of response. *Ann Rheum Dis* 1996; 55(11): 829-32.

59. Lambert RGW, Hutchings EJ, Grace MGA, Jhangri GS, Conner-Spady B, Maksymowych WP. Steroid injection for osteoarthritis of the hip - A randomized, double-blind, placebo-controlled trial. *Arthritis and Rheumatism* 2007; 56(7): 2278-87.

60. Meenagh GK, Patton J, Kynes C, Wright GD. A randomised controlled trial of intra-articular corticosteroid injection of the carpometacarpal joint of the thumb in osteoarthritis. *Ann Rheum Dis* 2004; 63(10): 1260-3.

61. Qvistgaard E, Christensen R, Torp-Pedersen S, Bliddal H. Intra-articular treatment of hip osteoarthritis: a randomized trial of hyaluronic acid, corticosteroid, and isotonic saline. *Osteoarthritis Cartilage* 2006; 14(2): 163-70.

62. Ravaud P, Moulinier L, Giraudeau B, et al. Effects of joint lavage and steroid injection in patients with osteoarthritis of the knee: results of a multicenter, randomized, controlled trial. *Arthritis Rheum* 1999; 42(3): 475-82.

63. Raynauld JP, Buckland-Wright C, Ward R, et al. Safety and efficacy of long - term intraarticular steroid injections in osteoarthritis of the knee: a randomized, double - blind, placebo - controlled trial. *Arthritis and Rheumatism*, 2003. http://onlinelibrary.wiley.com/o/cochrane/clcentral/articles/082/CN-00413082/frame.html (accessed.

64. Altman RD, Moskowitz R. Intraarticular sodium hyaluronate (Hyalgan) in the treatment of patients with osteoarthritis of the knee: A randomized clinical trial. *Journal of Rheumatology* 1998; 25(11): 2203-12.

65. Altman RD, Akermark C, Beaulieu AD, Schnitzer T. Efficacy and safety of a single intra-articular injection of non-animal stabilized hyaluronic acid (NASHA) in patients with osteoarthritis of the knee. *Osteoarthritis and Cartilage* 2004; 12(8): 642-9.

66. Altman RD, Rosen JE, Bloch DA, Hatoum HT, Korner P. A double-blind, randomized, saline-controlled study of the efficacy and safety of EUFLEXXA for treatment of painful osteoarthritis of the knee, with an open-label safety extension (the FLEXX trial). *Semin Arthritis Rheum* 2009; 39(1): 1-9.

67. Arden NK, Akermark C, Andersson M, Todman MG, Altman RD. A randomized saline-controlled trial of NASHA hyaluronic acid for knee osteoarthritis. *Current medical research and opinion* 2014; 30(2): 279-86.

68. Baltzer AW, Moser C, Jansen SA, Krauspe R. Autologous conditioned serum (Orthokine) is an effective treatment for knee osteoarthritis. *Osteoarthritis and cartilage / OARS, Osteoarthritis Research Society*, 2009. http://onlinelibrary.wiley.com/o/cochrane/clcentral/articles/579/CN-00702579/frame.html (accessed.

69. Carrabba M, Paresce E, Angelini M, Re KA, Torchiana EEM, Perbellini A. The safety and efficacy of different dose schedules of hyaluronic acid in the treatment of painful osteoarthritis of the knee with joint effusion. *European Journal of Rheumatology and Inflammation* 1995; 15(1): 25-31.

70. Chevalier X, Jerosch J, Goupille P, et al. Single, intra-articular treatment with 6 ml hylan G-F 20 in patients with symptomatic primary osteoarthritis of the knee: a randomised, multicentre, double-blind, placebo controlled trial. *Ann Rheum Dis* 2010; 69(1): 113-9.

71. Cohen MM, Altman RD, Hollstrom R, Hollstrom C, Sun C, Gipson B. Safety and efficacy of intra-articular sodium hyaluronate (Hyalgan (R)) in a randomized, double-blind study for osteoarthritis of the ankle. *Foot Ankle Int* 2008; 29(7): 657-63.

72. Corrado EM, Peluso GF, Gigliotti S, et al. The effects of intra-articular administration of hyaluronic acid on osteoarthritis of the knee: A clinical study with immunological and biochemical evaluations. *European Journal of Rheumatology and Inflammation* 1995; 15(1): 47-56.

73. Creamer P, Sharif M, George E, et al. Intra-articular hyaluronic acid in osteoarthritis of the knee: an investigation into mechanisms of action. *Osteoarthritis Cartilage* 1994; 2(2): 133-40.

74. Cubukcu D, Ardic F, Karabulut N, Topuz O. Hylan G-F 20 efficacy on articular cartilage quality in patients with knee osteoarthritis: clinical and MRI assessment. *Clin Rheumatol* 2005; 24(4): 336-41.

75. Day R, Brooks P, Conaghan PG, Petersen M. A Double Blind, Randomized, Multicenter, Parallel Group Study of the Effectiveness and Tolerance of Intraarticular Hyaluronan in Osteoarthritis of the Knee. *Journal of Rheumatology* 2004; 31(4): 775-82.

76. DeCaria JE, Montero-Odasso M, Wolfe D, Chesworth BM, Petrella RJ. The effect of intra-articular hyaluronic acid treatment on gait velocity in older knee osteoarthritis patients: A randomized, controlled study. *Archives of Gerontology and Geriatrics* 2012; 55(2): 310-5.

77. DeGroot H, 3rd, Uzunishvili S, Weir R, Al-omari A, Gomes B. Intra-articular injection of hyaluronic acid is not superior to saline solution injection for ankle arthritis: a randomized, double-blind, placebo-controlled study. *J Bone Joint Surg Am* 2012; 94(1): 2-8.

78. Dickson DJ, Hosie G, English JR. A double-blind, placebo-controlled comparison of hylan G-F 20 against diclofenac in knee osteoarthritis. *Journal of Clinical Research* 2001; 4(41-52): 41-52.

79. Diracoglu D, Vural M, Baskent A, Dikici F, Aksoy C. The effect of viscosupplementation on neuromuscular control of the knee in patients with osteoarthritis. *J Back Musculoskeletal Rehabil* 2009; 22(1): 1-9.

80. Dougados M, Nguyen M, Listrat V, Amor B. High molecular weight sodium hyaluronate (hyalectin) in osteoarthritis of the knee: a 1 year placebo-controlled trial. *Osteoarthritis Cartilage* 1993; 1(2): 97-103.

81. Grecomoro G, Martorana U, Di Marco C. Intra-articular treatment with sodium hyaluronate in gonarthrosis: a controlled clinical trial versus placebo. *Pharmatherapeutica* 1987; 5(2): 137-41.

82. Henderson EB, Smith EC, Pegley F, Blake DR. Intraarticular Injections of 750 Kd Hyaluronan in the Treatment of Osteoarthritis - a Randomized Single-Center Double-Blind Placebo-Controlled Trial of 91 Patients Demonstrating Lack of Efficacy. *Ann Rheum Dis* 1994; 53(8): 529-34.

83. Huang T-L, Chang C-C, Lee C-H, Chen S-C, Lai C-H, Tsai C-L. Intra-articular injections of sodium hyaluronate (Hyalgan[REGISTERED]) in osteoarthritis of the knee. a randomized, controlled, double-blind, multicenter trial in the Asian population. *BMC Musculoskelet Disord* 2011; 12: 221.

84. Huskisson EC, Donnelly S. Hyaluronic acid in the treatment of osteoarthritis of the knee. *Rheumatology (Oxford)* 1999; 38(7): 602-7.

85. Jubb RW, Piva S, Beinat L, Dacre J, Gishen P. A one-year, randomised, placebo (saline) controlled clinical trial of 500-730 kDa sodium hyaluronate (Hyalgan (R)) on the radiological change in osteoarthritis of the knee. *Int J Clin Pract* 2003; 57(6): 467-74.

86. Karlsson J, Sjogren LS, Lohmander LS. Comparison of two hyaluronan drugs and placebo in patients with knee osteoarthritis. A controlled, randomized, double-blind, parallel-design multicentre study. *Rheumatology (Oxford)* 2002; 41(11): 1240-8.

87. Kul-Panza E, Berker N. Is hyaluronate sodium effective in the management of knee osteoarthritis? A placebo-controlled double-blind study. *Minerva Med* 2010; 101(2): 63-72.

88. Lundsgaard C, Dufour N, Fallentin E, Winke P, Gluud C. Intra-articular sodium hyaluronate 2 mL versus physiological saline 20 mL versus physiological saline 2 mL for painful knee osteoarthritis: a randomized clinical trial. *Scand J Rheumatol* 2008; 37(2): 142-50.

89. Munteanu SE, Zammit GV, Menz HB, et al. Effectiveness of intra-articular hyaluronan (Synvisc, hylan G-F 20) for the treatment of first metatarsophalangeal joint osteoarthritis: a randomised placebo-controlled trial. *Ann Rheum Dis* 2011; 70(10): 1838-41.

90. Neustadt D, Caldwell J, Bell M, Wade J, Gimbel J. Clinical effects of intraarticular injection of high molecular weight hyaluronan (Orthovisc (R)) in osteoarthritis of the knee: A randomized, controlled, multicenter trial. *J Rheumatol* 2005; 32(10): 1928-36.

91. Petrella RJ, DiSilvestro MD, Hildebrand C. Effects of hyaluronate sodium on pain and physical functioning in osteoarthritis of the knee - A randomized, double-blind, placebo-controlled clinical trial. *Arch Intern Med* 2002; 162(3): 292-8.

92. Petrella RJ, Petrella M. A prospective, randomized, double-blind, placebo controlled study to evaluate the efficacy of intraarticular hyaluronic acid for osteoarthritis of the knee. *J Rheumatol* 2006; 33(5): 951-6.

93. Pham T, Henanff A, Ravaud P, Dieppe P, Paolozzi L, Dougados M. Evaluation of the symptomatic and structural efficacy of a new hyaluronic acid compound, NRD101, in comparison with diacerein and placebo in a 1 year randomised controlled study in symptomatic knee osteoarthritis. *Ann Rheum Dis*, 2004. http://onlinelibrary.wiley.com/o/cochrane/clcentral/articles/985/CN-00490985/frame.html (accessed.

94. Richette P, Ravaud P, Conrozier T, et al. Effect of hyaluronic acid in symptomatic hip osteoarthritis: a multicenter, randomized, placebo-controlled trial. *Arthritis Rheum* 2009; 60(3): 824-30.

95. Salk RS, Chang TJ, D'Costa WF, Soomekh DJ, Grogan KA. Sodium hyaluronate in the treatment of osteoarthritis of the ankle: a controlled, randomized, double-blind pilot study. *J Bone Joint Surg Am* 2006; 88(2): 295-302.

96. Scale D, Wobig M, Wolpert W. Viscosupplementation of Osteoarthritic Knees with Hylan - a Treatment Schedule Study. *Curr Ther Res-Clin Exp* 1994; 55(3): 220-32.

97. Sezgin M, Demirel AC, Karaca C, et al. Does hyaluronan affect inflammatory cytokines in knee osteoarthritis? *Rheumatol Int* 2005; 25(4): 264-9.

98. Kalunian KC, Moreland LW, Klashman DJ, et al. Visually-guided irrigation in patients with early knee osteoarthritis: a multicenter randomized, controlled trial. *Osteoarthritis and cartilage / OARS, Osteoarthritis Research Society*, 2000. http://onlinelibrary.wiley.com/o/cochrane/clcentral/articles/655/CN-00329655/frame.html (accessed.

99. Moseley JB, O'Malley K, Petersen NJ, et al. A controlled trial of arthroscopic surgery for osteoarthritis of the knee. *The New England journal of medicine*, 2002. http://onlinelibrary.wiley.com/o/cochrane/clcentral/articles/174/CN-00390174/frame.html (accessed.

100. Bradley JD, Heilman DK, Katz BP, Gsell P, Wallick JE, Brandt KD. Tidal irrigation as treatment for knee osteoarthritis: a sham-controlled, randomized, double-blinded evaluation. *Arthritis Rheum* 2002; 46(1): 100-8.

101. Baerwald C, Verdecchia P, Duquesroix B, Frayssinet H, Ferreira T. Efficacy, safety, and effects on blood pressure of naproxcinod 750 mg twice daily compared with placebo and naproxen 500 mg twice daily in patients with osteoarthritis of the hip: a randomized, double-blind, parallel-group, multicenter study. *Arthritis and rheumatism*, 2010. http://onlinelibrary.wiley.com/o/cochrane/clcentral/articles/856/CN-00777856/frame.html (accessed.

102. Bensen WG, Fiechtner JJ, McMillen JI, et al. Treatment of osteoarthritis with celecoxib, a cyclooxygenase-2 inhibitor: a randomized controlled trial. *Mayo Clinic Proceedings* 1999; 74(11): 1095-105.

103. Biegert C, Wagner I, Lüdtke R, et al. Efficacy and safety of willow bark extract in the treatment of osteoarthritis and rheumatoid arthritis: results of 2 randomized double-blind controlled trials. *Journal of Rheumatology* 2004; 31(11): 2121-30.

104. Bingham ICO, Sebba AI, Rubin BR, et al. Efficacy and safety of etoricoxib 30 mg and celecoxib 200 mg in the treatment of osteoarthritis in two identically designed, randomized, placebo-controlled, non-inferiority studies. *Rheumatology* 2007; 46(3): 496-507.

105. Bocanegra TS, Weaver AL, Tindall EA, et al. Diclofenac/misoprostol compared with diclofenac in the treatment of osteoarthritis of the knee or hip: a randomized, placebo controlled trial. Arthrotec Osteoarthritis Study Group. *The Journal of rheumatology*, 1998. http://onlinelibrary.wiley.com/o/cochrane/clcentral/articles/098/CN-00154098/frame.html (accessed.

106. Bourgeois P, Dreiser RL, Lequesne MG, Macciocchi A, Monti T. Multi-centre double-blind study to define the most favourable dose of nimesulide in terms of efficacy/safety ratio in the treatment of osteoarthritis. *European journal of rheumatology and inflammation*, 1994. http://onlinelibrary.wiley.com/o/cochrane/clcentral/articles/863/CN-00113863/frame.html (accessed.

107. Broell H, Tausch G. Clinical Effectiveness and Safety of Dexindoprofen Suppositories in Osteo Arthritis Patients a Double-Blind Placebo Controlled Trial. *Drugs under experimental and clinical research* 1984; 10(4): 277-82.

108. Case JP, Baliunas AJ, Block JA. Lack of efficacy of acetaminophen in treating symptomatic knee osteoarthritis: a randomized, double-blind, placebo-controlled comparison trial with diclofenac sodium. *Archives of Internal Medicine* 2003; 163(2): 169-78.

109. Cryer BL, Sostek MB, Fort JG, Svensson O, Hwang C, Hochberg MC. A fixed-dose combination of naproxen and esomeprazole magnesium has comparable upper gastrointestinal tolerability to celecoxib in patients with osteoarthritis of the knee: results from two randomized, parallel-group, placebo-controlled trials. *Annals of medicine*, 2011. http://onlinelibrary.wiley.com/o/cochrane/clcentral/articles/677/CN-00840677/frame.html (accessed.

110. Davies GM, Watson DJ, Bellamy N. Comparison of the responsiveness and relative effect size of the Western Ontario and McMaster Universities Osteoarthritis Index and the Short-Form Medical Outcomes Study Survey in a randomized, clinical trial of osteoarthritis patients. *Arthritis Care & Research* 1999; 12(3): 172-9.

111. Day R, Morrison B, Luza A, et al. A randomized trial of the efficacy and tolerability of the COX-2 inhibitor rofecoxib vs ibuprofen in patients with osteoarthritis. *Archives of Internal Medicine* 2000; 160(12): 1781-7.

112. DeLemos BP, Xiang J, Benson C, et al. Tramadol hydrochloride extended-release once-daily in the treatment of osteoarthritis of the knee and/or hip: a double-blind, randomized, dose-ranging trial. *American Journal of Therapeutics* 2011; 18(3): 216-26.

113. Dickson DJ, Hosie G, English JR. A double-blind, placebo-controlled comparison of hylan G-F 20 against diclofenac in knee osteoarthritis. *Journal of Drug Assessment* 2001; 4(Part 3): 179-90.

114. Dougados M, Moore A, Yu S, Gitton X. Evaluation of the patient acceptable symptom state in a pooled analysis of two multicentre, randomised, double-blind, placebo-controlled studies evaluating lumiracoxib and celecoxib in patients with osteoarthritis. *Arthritis research & therapy* 2007; 9(1): R11.

115. Dreiser RL, Gersberg M, Thomas F, Courcier S. Ibuprofen 800 mg for the treatment of osteoarthritis of the interphalangeal joints of the hand or trapezo metacarpal joint. *Revue du Rhumatisme (English Edition)* 1993; 60(11): 719-24.

116. Ehrich EW, Schnitzer TJ, McIlwain H, et al. Effect of specific COX-2 inhibition in osteoarthritis of the knee: A 6 week double blind, placebo controlled pilot study of rofecoxib. *Journal of Rheumatology* 1999; 26(11): 2438-47.

117. Ehrich EW, Bolognese JA, Watson DJ, Kong SX. Effect of rofecoxib therapy on measures of health-related quality of life in patients with osteoarthritis. *American Journal of Managed Care* 2001; 7(6): 609-16.

118. El-Mehairy MM, Shaker A, Bahgat NE, Hamza S, Salam MS. A double-blind comparison of niflumic acid with phenylbutazone, oxyphenylbutazone and placebo in the treatment of osteoarthrosis. *Rheumatol Rehabil* 1974; 13(4): 198-203.

119. Essex MN, O'Connell M, Bhadra Brown P. Response to nonsteroidal antiinflammatory drugs in African Americans with osteoarthritis of the knee. *Journal of International Medical Research* 2012; 40(6): 2251-66.

120. Fleischmann RM, Caldwell JR, Roth SH, Tesser JRP, Olson W, Kamin M. Tramadol for the treatment of joint pain associated with osteoarthritis: A randomized, double-blind, placebo-controlled trial. *Current Therapeutic Research-Clinical and Experimental* 2001; 62(2): 113-28.

121. Fleischmann R, Sheldon E, Maldonado-Cocco J, Dutta D, Yu S, Sloan VS. Lumiracoxib is effective in the treatment of osteoarthritis of the knee: a prospective randomized 13-week study versus placebo and celecoxib. *Clinical rheumatology*, 2005. http://onlinelibrary.wiley.com/o/cochrane/clcentral/articles/046/CN-00552046/frame.html (accessed.

122. Gibofsky A, Williams GW, McKenna F, Fort JG. Comparing the efficacy of cyclooxygenase 2-specific inhibitors in treating osteoarthritis: appropriate trial design considerations and results of a randomized, placebo-controlled trial. *Arthritis & Rheumatism* 2003; 48(11): 3102-11.

123. Gibofsky A, Hochberg MC, Jaros MJ, Young CL. Efficacy and safety of low-dose submicron diclofenac for the treatment of osteoarthritis pain: A 12 week, phase 3 study. *Current medical research and opinion* 2014; 30(9): 1883-93.

124. Goldstein DJ, Wang O, Todd LE, Gitter BD, DeBrota DJ, Iyengar S. Study of the analgesic effect of lanepitant in patients with osteoarthritis pain. *Clinical pharmacology and therapeutics*, 2000. http://onlinelibrary.wiley.com/o/cochrane/clcentral/articles/773/CN-00277773/frame.html (accessed.

125. Grifka JK, Zacher J, Brown JP, et al. Efficacy and tolerability of lumiracoxib versus placebo in patients with osteoarthritis of the hand. *Clinical and experimental rheumatology*, 2004. http://onlinelibrary.wiley.com/o/cochrane/clcentral/articles/194/CN-00492194/frame.html (accessed.

126. Haghighi M, Khalvat A, Toliat T, Jallaei S. Comparing the effects of ginger (Zingiber officinale) extract and ibuprofen on patients with osteoarthritis. *Archives of Iranian Medicine*, 2005. http://onlinelibrary.wiley.com/o/cochrane/clcentral/articles/391/CN-00557391/frame.html (accessed.

127. Karlsson J, Pivodic A, Aguirre D, Schnitzer TJ. Efficacy, safety, and tolerability of the cyclooxygenase-inhibiting nitric oxide donator naproxcinod in treating osteoarthritis of the hip or knee. *Journal of Rheumatology* 2009; 36(6): 1290-7.

128. Kivitz A, Eisen G, Zhao WW, Bevirt T, Recker DP. Randomized placebo-controlled trial comparing efficacy and safety of valdecoxib with naproxen in patients with osteoarthritis. *The Journal of family practice*, 2002. http://onlinelibrary.wiley.com/o/cochrane/clcentral/articles/059/CN-00390059/frame.html (accessed.

129. Kruger K, Klasser M, Mossinger J, Becker U. Oxaceprol--a randomised, placebo-controlled clinical study in osteoarthritis with a non-conventional non-steroidal anti-inflammatory drug. *Clin Exp Rheumatol* 2007; 25(1): 29-34.

130. Lee P, Davis P, Prat A. The efficacy of diflunisal in osteoarthritis of the knee. A Canadian multicenter study. *Journal of Rheumatology* 1985; 12(3): 544-8.

131. Leung AT, Malmstrom K, Gallacher AE, et al. Efficacy and tolerability profile of etoricoxib in patients with osteoarthritis: A randomized, double-blind, placebo and active-comparator controlled 12-week efficacy trial. *Current medical research and opinion* 2002; 18(2): 49-58.

132. Lund B, Distel M, Bluhmki E. A double-blind, randomized, placebo-controlled study of efficacy and tolerance of meloxicam treatment in patients with osteoarthritis of the knee.

Scandinavian Journal of Rheumatology, 1998. http://onlinelibrary.wiley.com/o/cochrane/clcentral/articles/400/CN-00720400/frame.html (accessed.

133. McKenna F, Borenstein D, Wendt H, Wallemark C, Lefkowith JB, Geis GS. Celecoxib versus diclofenac in the management of osteoarthritis of the knee: A placebo-controlled, randomised, double-blind comparison. *Scandinavian Journal of Rheumatology* 2001; 30(1): 11-8.

134. Nguyen M, Dougados M, Berdah L, Amor B. Diacerhein in the treatment of osteoarthritis of the hip. *Arthritis and rheumatism*, 1994. http://onlinelibrary.wiley.com/o/cochrane/clcentral/articles/253/CN-00100253/frame.html (accessed.

135. Paul S, Das N, Ghosh S. The effects of aceclofenac and nabumetone in osteoarthritis. *JNMA; journal of the Nepal Medical Association*, 2009. http://onlinelibrary.wiley.com/o/cochrane/clcentral/articles/226/CN-00753226/frame.html (accessed.

136. Pincus T, Koch G, Lei H, et al. Patient Preference for Placebo, Acetaminophen (paracetamol) or Celecoxib Efficacy Studies (PACES): Two randomised, double blind, placedo controlled, crossover clinical trials in patients with knee or hip osteoarthritis. *Annals of the Rheumatic Diseases* 2004; 63(8): 931-9.

137. Puopolo A, Boice JA, Fidelholtz JL, et al. A randomized placebo-controlled trial comparing the efficacy of etoricoxib 30 mg and ibuprofen 2400 mg for the treatment of patients with osteoarthritis. *Osteoarthritis and cartilage / OARS, Osteoarthritis Research Society*, 2007. http://onlinelibrary.wiley.com/o/cochrane/clcentral/articles/319/CN-00706319/frame.html (accessed.

138. Reginster Y, Malmstrom K, Mehta A, et al. Evaluation of the efficacy and safety of etoricoxib compared with naproxen in two, 138-week randomised studies of patients with osteoarthritis. *Annals of the Rheumatic Diseases* 2007; 66(7): 945-51.

139. Rother M, Lavins BJ, Kneer W, Lehnhardt K, Seidel EJ, Mazgareanu S. Efficacy and safety of epicutaneous ketoprofen in Transfersome (IDEA-033) versus oral cefecoxib and placebo in osteoarthritis of the knee: Multicentre randomised controlled trial. *Annals of the Rheumatic Diseases* 2007; 66(9): 1178-83.

140. Sandelin J, Harilainen A, Crone H, Hamberg P, Forsskahl B, Tamelander G. Local NSAID gel (Eltenac) in the treatment of osteoarthritis of the knee: A double blind study comparing eltenac with oral diclofenac and placebo gel. *Scandinavian Journal of Rheumatology* 1997; 26(4): 287-92.

141. Schmitt W, Walter K, Kurth HJ. Clinical trial on the efficacy and safety of different diclofenac formulations: Multiple-unit formulations compared to enteric coated tablets in patients with activated osteoarthritis. *Inflammopharmacology* 1999; 7(4): 363-75.

142. Schnitzer TJ, Beier J, Geusens P, et al. Efficacy and safety of four doses of lumiracoxib versus diclofenac in patients with knee or hip primary osteoarthritis: a phase II, four-week, multicenter, randomized, double-blind, placebo-controlled trial. *Arthritis & Rheumatism* 2004; 51(4): 549-57.

143. Schnitzer TJ, Dattani ID, Seriolo B, et al. A 13-week, multicenter, randomized, double-blind study of lumiracoxib in hip osteoarthritis. *Clinical Rheumatology* 2011; 30(11): 1433-46.

144. Schnitzer TJ, Hochberg MC, Marrero CE, Duquesroix B, Frayssinet H, Beekman M. Efficacy and safety of naproxcinod in patients with osteoarthritis of the knee: a 53-week prospective randomized multicenter study. *Seminars in Arthritis & Rheumatism* 2011; 40(4): 285-97.

145. Scott DL, Berry H, Capell H, et al. The long-term effects of non-steroidal anti-inflammatory drugs in osteoarthritis of the knee: a randomized placebo-controlled trial. *Rheumatology (Oxford, England)*, 2000. http://onlinelibrary.wiley.com/o/cochrane/clcentral/articles/598/CN-00331598/frame.html (accessed.

146. Sheldon E, Beaulieu A, Paster Z, Dutta D, Yu S, Sloan VS. Efficacy and tolerability of lumiracoxib in the treatment of osteoarthritis of the knee: a 13-week, randomized, double-blind comparison with celecoxib and placebo. *Clinical Therapeutics* 2005; 27(1): 64-77.

147. Shipley M, Berry H, Broster G, Jenkins M, Clover A, Williams I. Controlled trial of homoeopathic treatment of osteoarthritis. *Lancet* 1983; 1(8316): 97-8.

148. Simon LS, Grierson LM, Naseer Z, Bookman AAM, Zev Shainhouse J. Efficacy and safety of topical diclofenac containing dimethyl sulfoxide (DMSO) compared with those of topical placebo, DMSO vehicle and oral diclofenac for knee osteoarthritis. *Pain* 2009; 143(3): 238-45.

149. Svensson O, Malmenas M, Fajutrao L, Roos EM, Lohmander LS. Greater reduction of knee than hip pain in osteoarthritis treated with naproxen, as evaluated by WOMAC and SF-36. *Annals of the Rheumatic Diseases* 2006; 65(6): 781-4.

150. Tannenbaum H, Berenbaum F, Reginster JY, et al. Lumiracoxib is effective in the treatment of osteoarthritis of the knee: a 13 week, randomised, double blind study versus placebo and celecoxib. *Annals of the Rheumatic Diseases*, 2004. http://onlinelibrary.wiley.com/o/cochrane/clcentral/articles/129/CN-00489129/frame.html (accessed.

151. Truitt KE, Sperling RS, Ettinger WH, Jr., et al. A multicenter, randomized, controlled trial to evaluate the safety profile, tolerability, and efficacy of rofecoxib in advanced elderly patients with osteoarthritis. *Aging Clin* 2001; 13(2): 112-21.

152. Tuzun S, Uzun H, Aydin S, et al. Effects of flurbiprofen and tiaprofenic acid on oxidative stress markers in osteoarthritis: A prospective, randomized, open-label, active- and placebo-controlled trial. *Current Therapeutic Research-Clinical and Experimental* 2005; 66(4): 335-44.

153. Uzun H, Tuzun S, Ozaras N, et al. The effect of flurbiprofen and tiaprofenic acid on serum cytokine levels of patients with osteoarthrosis. *Acta Orthopaedica Scandinavica* 2001; 72(5): 499-502.

154. Wiesenhutter CW, Boice JA, Ko A, et al. Evaluation of the comparative efficacy of etoricoxib and ibuprofen for treatment of patients with osteoarthritis: A randomized, double-blind, placebo-controlled trial. *Mayo Clinic Proceedings* 2005; 80(4): 470-9.

155. Williams GW, Hubbard RC, Yu SS, Zhao W, Geis GS. Comparison of once-daily and twice-daily administration of celecoxib for the treatment of osteoarthritis of the knee. *Clinical Therapeutics* 2001; 23(2): 213-27.

156. Wittenberg RH, Schell E, Krehan G, et al. First-dose analgesic effect of the cyclo-oxygenase-2 selective inhibitor lumiracoxib in osteoarthritis of the knee: a randomized, double-blind, placebo-controlled comparison with celecoxib [NCT00267215]. *Arthritis research & therapy* 2006; 8(2): R35.

157. Altman RD, Zinsenheim JR, Temple AR, Schweinle JE. Three-month efficacy and safety of acetaminophen extended-release for osteoarthritis pain of the hip or knee: a randomized, double-blind, placebo-controlled study. *Osteoarthritis and cartilage / OARS, Osteoarthritis Research Society*, 2007. http://onlinelibrary.wiley.com/o/cochrane/clcentral/articles/291/CN-00586291/frame.html (accessed.

158. Golden HE, Moskowitz RW, Minic M. Analgesic efficacy and safety of nonprescription doses of naproxen sodium compared with acetaminophen in the treatment of osteoarthritis of the knee. *American journal of therapeutics*, 2004. http://onlinelibrary.wiley.com/o/cochrane/clcentral/articles/214/CN-00469214/frame.html (accessed.

159. Miceli-Richard C, Le Bars M, Schmidely N, Dougados M. Paracetamol in osteoarthritis of the knee. *Annals of the rheumatic diseases* 2004; 63(8): 923-30.

160. Atamaz FC, Durmaz B, Baydar M, et al. Comparison of the efficacy of transcutaneous electrical nerve stimulation, interferential currents, and shortwave diathermy in knee osteoarthritis: a double-blind, randomized, controlled, multicenter study. *Archives of physical medicine and rehabilitation*, 2012. http://onlinelibrary.wiley.com/o/cochrane/clcentral/articles/724/CN-00880724/frame.html (accessed.

161. Ay S, Evcik D. The effects of pulsed electromagnetic fields in the treatment of knee osteoarthritis: a randomized, placebo-controlled trial. *Rheumatology international*, 2009. http://onlinelibrary.wiley.com/o/cochrane/clcentral/articles/928/CN-00697928/frame.html (accessed.

162. Callaghan MJ, Whittaker PE, Grimes S, Smith L. An evaluation of pulsed shortwave on knee osteoarthritis using radioleucoscintigraphy: a randomised, double blind, controlled trial. *Joint, bone, spine*, 2005. http://onlinelibrary.wiley.com/o/cochrane/clcentral/articles/266/CN-00521266/frame.html (accessed.

163. Fischer G, Pelka RB, Barovic J. Adjuvant treatment of osteo arthritis of the knee with weak pulsing magnetic fields. Results of a prospective, placebo controlled trial. [German]

Adjuvante behandlung der gonarthrose mit schwachen pulsierenden magnetfeldern: Ergebnisse einer prospektiven, plazebo-kontrollierten vergleichenden therapiestudie. *Zeitschrift fur Orthopadie und ihre Grenzgebiete* 2005; 143(5): 544-50.

164. Fukuda TY, Ovanessian V, Cunha RA, et al. Pulsed short wave effect in pain and function in patients with knee osteoarthritis. *Journal of applied research* 2008; 8(3): 189-98.

165. Fukuda TY, Alves da Cunha R, Fukuda VO, et al. Pulsed shortwave treatment in women with knee osteoarthritis: a multicenter, randomized, placebo-controlled clinical trial. *Physical therapy* 2011; 91(7): 1009-17.

166. Kanat E, Alp A, Yurtkuran M. Magnetotherapy in hand osteoarthritis: A pilot trial. *Complementary therapies in medicine*, 2013. http://onlinelibrary.wiley.com/o/cochrane/clcentral/articles/942/CN-00910942/frame.html (accessed.

167. Klaber Moffett JA, Richardson PH, Frost H, Osborn A. A placebo controlled double blind trial to evaluate the effectiveness of pulsed short wave therapy for osteoarthritic hip and knee pain. *Pain* 1996; 67(1): 121-7.

168. Lee J, Park J, DH S, et al. Effect of pulsed electromagnetic fields in the treatment of knee osteoarthritis. Report of double-blind, placebo-controlled, randomized trial. *J Korean Rheum Assoc* 2004; 11: 143-50.

169. Nelson FR, Zvirbulis R, Pilla AA. Non-invasive electromagnetic field therapy produces rapid and substantial pain reduction in early knee osteoarthritis: A randomized double-blind pilot study. *Rheumatology international*, 2013. http://onlinelibrary.wiley.com/o/cochrane/clcentral/articles/418/CN-00919418/frame.html (accessed.

170. Nicolakis P, Kollmitzer J, Crevenna R, Bittner C, Erdogmus CB, Nicolakis J. Pulsed magnetic field therapy for osteoarthritis of the knee - A double-blind sham-controlled trial. *Wiener klinische Wochenschrift* 2002; 114(15-16): 678-84.

171. Ozguclu E, Cetin A, Cetin M, Calp E. Additional effect of pulsed electromagnetic field therapy on knee osteoarthritis treatment: A randomized, placebo-controlled study. *Clinical rheumatology* 2010; 29(8): 927-31.

172. Pipitone N, Scott DL. Magnetic pulse treatment for knee osteoarthritis: a randomised, double-blind, placebo-controlled study. *Current medical research and opinion*, 2001. http://onlinelibrary.wiley.com/o/cochrane/clcentral/articles/691/CN-00388691/frame.html (accessed.

173. Rattanachaiyanont M, Kuptniratsaikul V. No additional benefit of shortwave diathermy over exercise program for knee osteoarthritis in peri-/post-menopausal women: an equivalence trial. *Osteoarthritis and Cartilage* 2008; 16(7): 823-8.

174. Sutbeyaz ST, Sezer N, Koseoglu BF. The effect of pulsed electromagnetic fields in the treatment of cervical osteoarthritis: a randomized, double-blind, sham-controlled trial. *Rheumatology international*, 2006. http://onlinelibrary.wiley.com/o/cochrane/clcentral/articles/824/CN-00560824/frame.html (accessed.

175. Thamsborg G, Floresu A, Oturai P, Fallentin E, Tritsaris K, Dissing S. Treatment of knee osteoarthritis with pulsed electromagnetic fields: A randomized, double-blind, placebo-controlled study. *Osteoarthritis and cartilage* 2005; 13(7): 575-81.

176. Trock DH, Bollet AJ, Dyer Jr RH, Fielding LP, Miner WK, Markoll R. A double-blind trial of the clinical effects of pulsed electromagnetic fields in osteoarthritis. *Journal of rheumatology* 1993; 20(3): 456-60.

177. Trock DH, Bollet AJ, Markoll R. The Effect of Pulsed Electromagnetic-Fields in the Treatment of Osteoarthritis of the Knee and Cervical-Spine - Report of Randomized, Double-Blind, Placebo-Controlled Trials. *Journal of rheumatology* 1994; 21(10): 1903-11.

178. Allegrini A, Nuzzo L, Pavone D, et al. Efficacy and safety of piroxicam patch versus piroxicam cream in patients with lumbar osteoarthritis: A randomized, placebo-controlled study. *Arzneimittel-Forschung/Drug Research* 2009; 59(8): 403-9.

179. Altman RD, Dreiser R-L, Fisher CL, Chase WF, Dreher DS, Zacher J. Diclofenac sodium gel in patients with primary hand osteoarthritis: a randomized, double-blind, placebo-controlled trial. *Journal of Rheumatology* 2009; 36(9): 1991-9.

180. Baer PA, Thomas LM, Shainhouse Z. Treatment of osteoarthritis of the knee with a topical diclofenac solution: a randomised controlled, 6-week trial [ISRCTN53366886]. *BMC Musculoskeletal Disorders*, 2005. http://onlinelibrary.wiley.com/o/cochrane/clcentral/articles/372/CN-00529372/frame.html (accessed.

181. Baraf HSB, Gold MS, Clark MB, Altman RD. Safety and efficacy of topical diclofenac sodium 1% gel in knee osteoarthritis: a randomized controlled trial. *Physician and Sportsmedicine*, 2010. http://onlinelibrary.wiley.com/o/cochrane/clcentral/articles/187/CN-00803187/frame.html (accessed.

182. Bookman AAM, Williams KSA, Shainhouse JZ. Effects of a tropical diclofenac solution for relieving symtoms of primary osteoarthritis of the knee: A randomized controlled trial. *Cmaj* 2004; 171(4): 333-8.

183. Bruhlmann P, Michel BA. Topical diclofenac patch in patients with knee osteoarthritis: A randomized, double-blind, controlled clinical trial. *Clinical and experimental rheumatology* 2003; 21(2): 193-8.

184. Conaghan PG, Dickson J, Bolten W, Cevc G, Rother M. A multicentre, randomized, placebo- and active-controlled trial comparing the efficacy and safety of topical ketoprofen in Transfersome gel (IDEA-033) with ketoprofen-free vehicle (TDT 064) and oral celecoxib for knee pain associated with osteoarthritis. *Rheumatology (Oxford, England)*, 2013. http://onlinelibrary.wiley.com/o/cochrane/clcentral/articles/886/CN-00876886/frame.html (accessed.

185. Dreiser RL, Tisne-Camus M. DHEP plasters as a topical treatment of knee osteoarthritis--a double-blind placebo-controlled study. *Drugs Exp Clin Res* 1993; 19(3): 117-23.

186. Grace D, Rogers J, Skeith K, Anderson K. Topical diclofenac versus placebo: a double blind, randomized clinical trial in patients with osteoarthritis of the knee. *Journal of Rheumatology* 1999; 26(12): 2659-63.

187. Niethard FU, Gold MS, Solomon GS, et al. Efficacy of topical diclofenac diethylamine gel in osteoarthritis of the knee. *Journal of Rheumatology* 2005; 32(12): 2384-92.

188. Ottillinger B, Gomor B, Michel BA, Pavelka K, Beck W, Elsasser U. Efficacy and safety of eltenac gel in the treatment of knee osteoarthritis. *Osteoarthritis and Cartilage* 2001; 9(3): 273-80.

189. Roth SH. A controlled clinical investigation of 3% diclofenac/2.5% sodium hyaluronate topical gel in the treatment of uncontrolled pain in chronic oral NSAID users

with osteoarthritis. *International journal of tissue reactions*, 1995. http://onlinelibrary.wiley.com/o/cochrane/clcentral/articles/301/CN-00731301/frame.html (accessed.

190. Roth SH, Shainhouse JZ. Efficacy and safety of a topical diclofenac solution (Pennsaid) in the treatment of primary osteoarthritis of the knee: a randomized, double-blind, vehicle-controlled clinical trial. *Archives of Internal Medicine* 2004; 164(18): 2017-23.

191. Rother M, Conaghan PG. A randomized, double-blind, phase III trial in moderate osteoarthritis knee pain comparing topical ketoprofen gel with ketoprofen-free gel. *J Rheumatol* 2013; 40(10): 1742-8.

192. Rovensky J, Micekova D, Gubzova Z, et al. Treatment of knee osteoarthritis with a topical non-steroidal antiinflammatory drug. Results of a randomized, double-blind, placebo-controlled study on the efficacy and safety of a 5% ibuprofen cream. *Drugs under experimental and clinical research* 2001; 27(5-6): 209-21.

193. Trnavsky K, Fischer M, Vogtle-Junkert U, Schreyger F. Efficacy and safety of 5% ibuprofen cream treatment in knee osteoarthritis. Results of a randomized, double-blind, placebo-controlled study. *Journal of Rheumatology* 2004; 31(3): 565-72.

194. Varadi G, Zhu Z, Blattler T, et al. Randomized clinical trial evaluating transdermal ibuprofen for moderate to severe knee osteoarthritis. *Pain physician*, 2013. http://onlinelibrary.wiley.com/o/cochrane/clcentral/articles/873/CN-00961873/frame.html (accessed.
